# Comparative Phosphoproteomic Analysis under High-Nitrogen Fertilizer Reveals Central Phosphoproteins Promoting Wheat Grain Starch and Protein Synthesis

**DOI:** 10.3389/fpls.2017.00067

**Published:** 2017-01-30

**Authors:** Shoumin Zhen, Xiong Deng, Ming Zhang, Gengrui Zhu, Dongwen Lv, Yaping Wang, Dong Zhu, Yueming Yan

**Affiliations:** ^1^College of Life Science, Capital Normal UniversityBeijing, China; ^2^College of Life Science, Heze UniversityShandong, China; ^3^Hubei Collaborative Innovation Center for Grain IndustryJingzhou, China

**Keywords:** *Triticum aestivum* L., phosphoproteome, grain development, high nitrogen stress, starch biosynthesis, breadmaking quality

## Abstract

Nitrogen (N) is a macronutrient important for plant growth and development. It also strongly influences starch and protein synthesis, closely related to grain yield and quality. We performed the first comparative phosphoproteomic analysis of developing wheat grains in response to high-N fertilizer. Physiological and biochemical analyses showed that application of high-N fertilizer resulted in significant increases in leaf length and area, chlorophyll content, the activity of key enzymes in leaves such as nitrate reductase (NR), and in grains such as sucrose phosphate synthase (SPS), sucrose synthase (SuSy), and ADP glucose pyrophosphorylase (AGPase). This enhanced enzyme activity led to significant improvements in starch content, grain yield, and ultimately, bread making quality. Comparative phosphoproteomic analysis of developing grains under the application of high-N fertilizer performed 15 and 25 days post-anthesis identified 2470 phosphosites among 1372 phosphoproteins, of which 411 unique proteins displayed significant changes in phosphorylation level (>2-fold or <0.5-fold). These phosphoproteins are involved mainly in signaling transduction, starch synthesis, energy metabolism. Pro-Q diamond staining and Western blotting confirmed our phosphoproteomic results. We propose a putative pathway to elucidate the important roles of the central phosphoproteins regulating grain starch and protein synthesis. Our results provide new insights into the molecular mechanisms of protein phosphorylation modifications involved in grain development, yield and quality formation.

## Introduction

Wheat is one of the “big three” cereal crops, with over 600 million tons harvested annually (Shewry, 2009). The mature wheat grain comprises three major components: starch, proteins, and cell wall polysaccharides, in addition to various minor components such as lipids, terpenoids, phenolics, minerals, and vitamins (Shewry et al., 2013). Wheat crops are largely used for human food and livestock feed.

Nitrogen (N) is one of the major plant nutrients limiting crop production worldwide. Because plants require more N than any other mineral element, N deficiency is a limiting factor for plant growth (Krouk et al., 2010). In plants, nitrate has been shown to serve both as a nutrient and signaling metabolite, having profound effects on both plant metabolism and growth (Glass, 2003). N is also required for the synthesis of amino acids, proteins, chlorophyll, nucleic acids, lipids, and a variety of other metabolites whose structure contains N. Plants take up nitrate from the soil via specific transporters, and then nitrate is reduced to ammonium by the concerted actions of nitrate reductase and nitrite reductase. Subsequently, the assimilated N is used to produce amino acids, while carbon dioxide is fixed to synthesize sugars (Urbanczyk-Wochniak and Fernie, 2005). Nitrate levels can reprogram carbohydrate metabolism, during nitrate assimilation, carbohydrate synthesis is decreased, and more carbon is converted into organic acids (Stitt et al., 2002). Most signaling pathways involve modulation of protein abundance and/or activity via protein phosphorylation (Engelsberger and Schulze, 2012). However, the changes in protein phosphorylation induced by N remain poorly understood.

Protein post-translational modifications (PTMs) are closely related to plant growth, development, and resistance to various biotic and abiotic stresses. To date, more than 461 distinct PTMs have been described (Khoury et al., 2011), and it is increasingly clear that many, perhaps most, proteins are decorated with multiple PTMs (Hunter, 2007). Particularly, phosphorylation is one of the most common and important modifications; reversible and often transient, phosphorylation regulates essential molecular events in the cell cycle, DNA transcription, energy metabolism, and important biological processes including seed germination, stomatal movement, innate immune response and defense, and stress tolerance (Kersten et al., 2009). Since phosphorylated proteins/peptides are often of high heterogeneity and low stoichiometry in a biological sample, it is imperative to enrich phosphorylated peptides or proteins prior to MS analysis (Silva-Sanchez et al., 2015). In recent years, several techniques have been developed for specific enrichment of phosphopeptides or phosphoproteins, such as TiO_2_–metal oxide affinity chromatography, immunoaffinity enrichment, immobilized metal affinity chromatography, hydrophilic interaction liquid chromatography, Phos-Tag chromatography, prefractionation by ion exchange chromatography and electrostatic repulsion hydrophilic interaction chromatography, polymer-based metal ion affinity capture. These technologies provide powerful tools for different aspects of phosphoproteomic research. TiO_2_–metal oxide affinity chromatography has been widely used because of its high sensitivity. Protein phosphorylation is one of the major mechanisms involved in stress signal transmission (Rampitsch and Bykova, 2012); extensive phosphoproteomic studies have been performed in plants, such as wheat under various abiotic stress (Zhang et al., 2014a,b), maize (Hu et al., 2015a,b; Vu et al., 2016), rice (Chang et al., 2012), barley (Horie et al., 2011), diploid wheat (*Triticum monococcum*, Lv et al., 2016), and *Brachypodium distachyon* L. (Lv et al., 2014a). To date, only two studies related to N phosphoproteomics in *Arabidopsis* have been reported. Engelsberger and Schulze (2012) found that N resupply in the form of ammonium or nitrate resulted in distinct phosphorylation patterns, and these phosphoproteins were mainly associated with transporters, signaling functions, and transcription factors. Using the Pro-Q staining approach, Wang et al. (2012) identified 38 proteins with significant changes in phosphorylation status in plants deprived of N for up to 48 h followed by a 24 h recovery period. However, to our knowledge, the regulatory mechanisms underlying the effects of phosphorylation modification on wheat grain development, yield and quality under high N conditions are still not clear.

In this work, we performed the first comparative phosphoproteomic analysis of developing wheat grains (15 and 25 days post-anthesis, DPA) under high N fertilization using TiO_2_ enrichment, liquid chromatography/tandem mass spectrometry (LC-MS/MS) analysis and label-free phosphopeptide quantification. Our results provide new insights from a phosphoproteomics perspective into the regulatory mechanisms of grain development and yield, quality formation in response to high N stress.

## Materials and methods

### Wheat materials, field treatments and sampling

Chinese elite winter wheat cultivar “Zhongmai 175” (*Triticum aestivum* L.) was used as material and grown in the experimental station of China Agricultural University, Wuqiao, Hebei Province (116°37′23″E, 37°16′02″N) during the 2014–2015 growing season. The recent studies have showed that high-N fertilizer urea (NH_2_)_2_CO of 240 kg/hm^2^ promotes grain yield and protein content (Li et al., 2016). Thus, 240 kg/hm^2^ urea was used as high-N treatment. Field experiment included a control group with normal N fertilization of 180 kg/hm^2^ and a treatment group with high-N fertilization of 240 kg/hm^2^. The experimental design, N application and cultivation were same as our recent report (Zhen et al., 2016). The flag leaves and developing grains from five periods (10, 15, 20, 25, and 30 days post-anthesis, DPA) in three biological replicates were collected and used for measuring physiological and biochemical parameters. Developing grains at 15 and 25 DPA were used for protein extraction and phosphoproteomic analysis. All samples were quickly collected and immediately placed in liquid nitrogen, and then stored at −80°C prior to analysis.

### Measurements of physiological and biochemical parameters, and agronomic traits

Some important physiological and biochemical parameters and agronomic traits at five different time points after flowering in both groups were measured. Chlorophyll content determination were according to the previous reports (Lv et al., 2014a; Zhang et al., 2014a). The activity of NR in flag leaves, SPS, SuSy, and AGPase of wheat grains were measured by using the kit (Cat. No IY1, SPS-1-Y, SS-1-Y, and AGP-1-Y) supplied by Suzhou Keming science and technology co., Ltd. (China). According to the absorances at different wavelength, we calculated the activities of these enzymes. The flag leaf area and length were measured by the Li-3000C leaf area meter. Total starch content was tested using total starch assay kits (Megazyme Int. Ireland, Ireland) according to the manufacturer's protocols and Chen et al. (2016). The main agronomic traits of the mature plants were recorded, including plant height, ear length, number of spikelets, number of infertile spikelets, kernels per spike, weight of a thousand kernels, and grain yield. Statistical analyses were conducted using independent Student's *t*-tests with SPSS statistics software (version 19.0).

### Grain microstructure observation

Chemical fixation for grain microstructural observation was based on Guillon et al. (2011) with minor modifications. Light microscopy and SEM observation was performed following our previous study (Chen et al., 2014).

### SE-HPLC

Size-exclusion high performance liquid chromatography (SE-HPLC) was used to separate and quantify the gluten macropolymer (GMP) contents according to Rakszegia et al. (2008) and Wang et al. (2013).

### Protein extraction

Proteins were extracted with the same procedures of previous study in our lab (Zhang et al., 2014a) with minor modifications. Grain samples of 0.5 g were ground into fine powder in liquid nitrogen, and then these powders were mixed with 1 mL of extraction buffer (50 mM Tris-HCl, pH 8.0, 0.1 M KCl, 5 mM EDTA, 30% sucrose) containing PhosSTOP phosphatase inhibitor cocktail (1 tablet/10 mL; Roche, Basel, Switzerland), followed by placement for 15 min in extraction buffer. After that, these samples were shaken vigorously for 30 min at room temperature. These samples were centrifuged for two times, protein supernatants were precipitated with a one-quarter volume of cold 10% trichloroacetic acid at −20°C for 4 h. Then centrifuging, the pellets were rinsed with cold (−80°C) acetone and then centrifuged three times at 13,000 g for 10 min. These pellets were freeze dried and added to 300 μL of solubilization buffer at room temperature for 4 h. After insoluble material was removed, the concentrations of protein samples were determined with a 2-D Quant Kit (Amersham Bioscience, USA) according to the protocol, and the final protein solution was stored at −80°C for later use. Extracted proteins from each biological replicate were adjusted to the same concentration for the following analysis.

### Phosphopeptide enrichment using TiO_2_ microcolumns

These extracted protein samples were directly reduced with dithiothreitol (DTT), alkylated with iodoacetamide, and digested with endoproteinase Lys-C and trypsin, as previous study (Olsen et al., 2006). The enrichment for the phosphopeptide procedure was performed in accordance with Lv et al. (2014b), with some minor modifications. TiO_2_ beads (GL Sciences, Tokyo, Japan) were incubated in 400 μL of loading buffer containing 65% acetonitrile (ACN)/2% trifluoroacetic acid (TFA) saturated with glutamic acid. Then, 2 mg of tryptic peptides was dissolved with 550 μL of loading buffer and incubated with the appropriate amount of TiO_2_ beads. After washing with 600 μL of wash buffer (65% ACN/0.1% TFA), the phosphopeptides were eluted twice with 300 μL of elution buffer (500 mM NH_4_OH/60% ACN). The eluates were dried and reconstituted in 0.1% formic acid (FA)/H_2_O for the following analysis.

### Phosphopeptide identification and phosphorylation site localization

The enriched phosphopeptides were separated on a self-packed C18 reversed-phase column (70 μm inner diameter, 150 mm length) (ColumnTechnology Innovation CTI, Fremont, CA) that was directly connected with a nanoelectrospray ion source in an LTQ-Orbitrap XL mass spectrometer (Thermo Fisher Scientific, America) according to Zhang et al. (2014a). The mobile phases consisted of 0.1% FA (A) and 0.1% FA and 90% ACN (B). A five-step linear gradient of 5–30% B in 105 min, 30–90% B in 16 min, 90% B for 4 min, 90–2% B in 0.5 min, and 2% B for 14.5 min was employed. The spray voltage was set to 2.0 kV, and the temperature of the heated capillary was set at 240°C. All of other conditions were in accordance with Zhang et al. (2014a) and Lv et al. (2014b). Three biological replicates of each sample were used for the identification independently. The raw files were processed using MaxQuant (version 1.3.0.5) and were compared with the wheat database (77037 entries, as described by Lv et al., 2016). Up to two missing cleavage points were allowed. The precursor ion mass tolerance was 7 ppm, and the fragment ion mass tolerance was 0.5 Da for the MS/MS spectra. The false discovery rate (FDR) was set to < 1.0% for the identification of both peptides and proteins. The minimum peptide length was set to 6. Potential phosphorylation residues were then grouped into three categories depending on their PTM localization probabilities: class I (localization probability, *P* ≥ 0.75), class II (0.75 > *P* ≥ 0. 5), and class III (*P* < 0.5). An FDR of 1% was used to identify phosphorylation residues. Spectra without residue-determining ions were used to identify phosphopeptides with undetermined residues.

### Verification of phosphoproteins by Pro-Q diamond gel staining and western blotting

The 2D gels were isoelectric focused firstly. Isoelectric focusing of each sample was performed as follows: active rehydration was carried out at 30 V for 12 h, followed by 300 V for 1 h, 500 V for 1 h, 1000 V for 1 h, 3000 V for 1 h, and then focusing at 8000 V until 80,000 Vh at 20°C. After that, the strips were equilibrated with an equilibration solution containing 1% DTT for 15 min, and a second equilibration step of 15 min containing 2.5% w/v iodoacetamide. The equilibrated strips were loaded on the top of 12% SDS-polyacrylamide gels and sealed with 0.5% w/v agarose. The SDS-PAGE step was performed at 15°C in an electrophoresis system at a constant current setting of 15 mA/gel for 1 h, followed by 20 mA/gel until the bromphenol blue tracking dye arrived at the bottom edge of the gel, these protocols were consistent with Lv et al. (2014a). After that, 2D gels were stained with Pro-Q Diamond (Invitrogen, USA) based on the manufacturer's instructions and Zhang et al. (2014a). Anti-Phosphoserine/threonine/tyrosine monoclonal antibody was bought from Abcam (Cat. No. SPM101, MA, USA). Western blotting was performed following our previous study (Chen et al., 2014).

### Bioinformatic analyses

The Blast2GO software were used to perform gene ontology (GO) annotation (http://www.blast2go.com/b2ghome) according to Conesa and Götz (2008). Significantly enriched phosphorylation motifs were extracted from phosphopeptides with confidently identified phosphorylation residues using the Motif-X algorithm (http://motif-x.med.harvard.edu/) (Meyer et al., 2012). The phosphoproteins blasted by the NCBI were used to obtain the EuKaryotic Orthologous Groups (KOG) numbers of those proteins by eggNOG (http://eggnog.embl.de/version_3.0/). A data set containing all the KOG numbers was then used for protein-protein interaction (PPI) analysis by using the Search Tool for Retrieval of Interacting Genes/Proteins (STRING) database (http://string-db.org/). Only the phosphoproteins that had a high confidence score of at least 0.9 and were based on coexpression and experiment conditions were used to construct the network. Then, they were displayed using Cytoscape (version 3.0) software. NetSurfP (http://www.cbs.dtu.dk/services/NetSurfP/) (Bent et al., 2008) and Phyre2 (http://www.sbg.Bio.ic.ac.uk/phyre2/html/page.cgi?id=index) (Kelley and Sternberg, 2009) were used to predict the two and three dimensional structures of certain phosphoproteins, respectively. The phosphorylated residues were displayed using Swiss-Pdb Viewer (SPDBV) software (http://spdbv.vital-it.ch/).

## Results

The experimental design of this study is shown in Figure S1. Field experiment treatments included a control group (180 kg/hm^2^ urea) and a high-N treatment group (240 kg/hm^2^ urea) with three replicates. Important physiological and biochemical parameters, agronomic traits, grain microstructure, and bread making quality were measured at different developmental stages (10, 15, 20, 25, and 30 DPA). Subsequently, developing grains at 15 and 25 DPA were used for protein extraction and phosphoproteomic analysis via TiO_2_ enrichment, LC-MS/MS, label-free phosphopeptide quantification and bioinformatics analysis. Pro-Q Diamond staining and Western blotting using an anti-phosphoserine/threonine/tyrosine monoclonal antibody were then used to confirm the phosphoproteomic results.

### Plant phenotype and physiological, biochemical and agronomic trait changes under high-N treatment

Generally, leaves became greener and plant growth more vigorous after high-N application. Compared with the control group, the high-N treatment group experienced significant changes in plant growth and developmental features, physiological and biochemical characteristics, main agronomic traits and yield performance (Figure S2).

**Figure 1:**
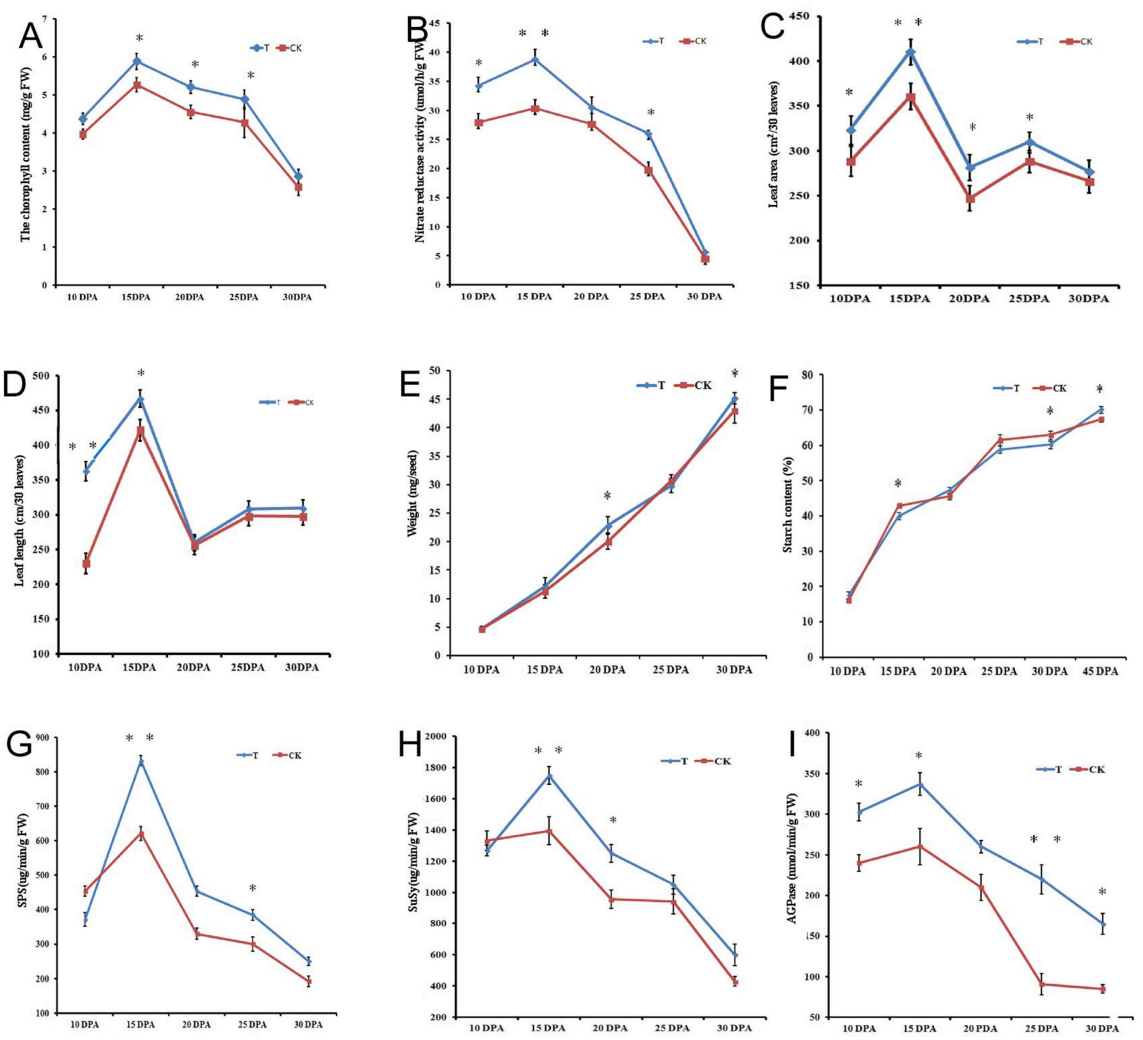
**Physiological and biochemical changes of flag leaves (A–D)** and grains **(E–I)** from Chinese elite bread wheat cultivar Zhongmai 175 under high N and normal N fertilizer conditions. **(A)** Total chlorophyll content. **(B)** Nitrate reducase activity. **(C)** Leaf area. **(D)** Leaf length. **(E)** Grain weight. **(F)** Total starch content. **(G)** SPS activity. **(H)** SuSy activity. **(I)** AGPase activity. Error bars indicate standard errors of three biological replicates. Statistically significant differences between treatments and the control were calculated by independent Student's *T*-tests: ^*^p < 0.05; ^**^ *p* < 0.01. T and CK indicate the high-N treated group and control group, respectively.

Physiological and biochemical analysis of flag leaves (Figures 1A–D) and seeds (Figures 1E–F) showed significant differences between the two N-treated groups. Total chlorophyll content at 15 DPA increased sharply in both groups and then decreased gradually until 30 DPA. At 15, 20, and 25 DPA, there were significant differences (*p* < 0.05) in total chlorophyll content between the groups (Figure 1A). NR in both groups showed a similar trend during seed development. At 15 DPA, there was a significant difference in NR content (*p* < 0.01) between treatments, and NR content decreased steadily from 20 to 30 DPA (Figure 1B). Flag leaf area and length were significantly increased under the high-N treatment, particularly during the early grain developmental stages (Figures 1C–D). Grain weight increased gradually from 10 to 30 DPA, and the N-treated group had a higher mass than that of the control group, with a sharp increase at 20 and 30 DPA (Figure 1E). Starch content testing during grain development showed that the high-N treatment improved starch biosynthesis; starch content at grain maturation was significantly increased, from 67.3% (control group) to 70.1% (high N group) (Figure 1F). In particular, starch content increased by 10% from 30 to 45 DPA after high-N fertilization, but that in the control group increased by only 5% over the same period. Activities of the starch biosynthesis-related enzymes SPS, SuSy, and AGPase were also significantly improved after high-N treatment (Figures 1G–I).

Agronomic and yield trait analyses showed that the high-N treatment significantly increased plant height, kernels per spike, effective spikelet number, and grain weight/spike, ultimately resulting in a 5.3% increase in grain yield (Figure S2).

### Grain microstructure and quality changes under high-N treatment

**Figure 2:**
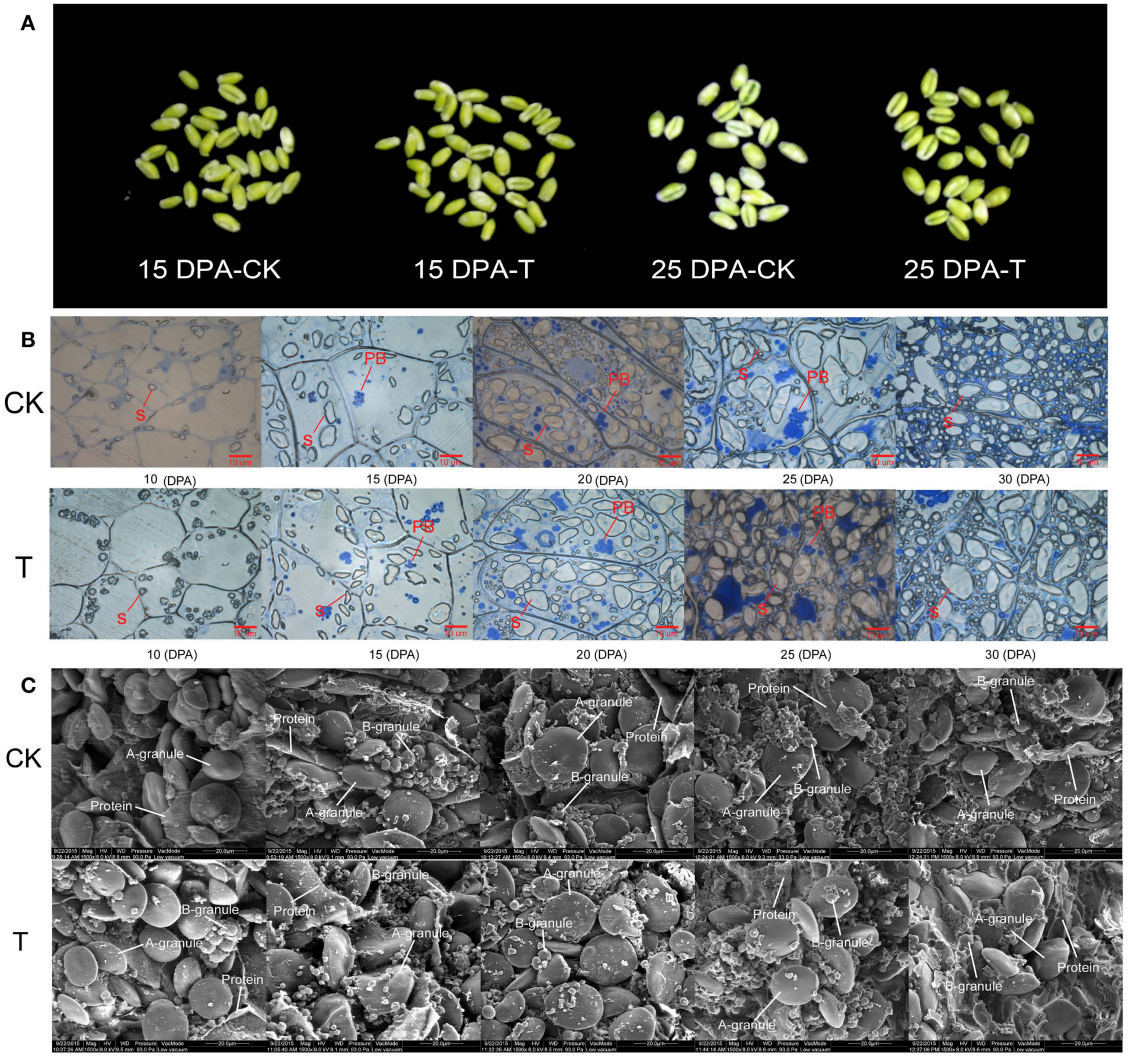
**Grain phenotype and microstructure changes under high N treatment (T) and normal N fertilization (CK). (A)** Grain appearance at 15 and 25 DPA under different N fertilizer conditions. **(B)** Light microscope images of developing grains from 5 periods in CK and high N treated groups. The scale bar is 10 μm. The starch and protein body (PB) are marked with lines. **(C)** SEM images of developing grains from 5 periods in CK and high N treated groups. The scale bar is 20 μm. The **A** and **B** starch granules are marked with arrows.

When the high-N fertilizer was applied, the grain filling period was extended, and grain morphology showed significant changes (Figure 2A). We examined dynamic accumulation patterns of starch granules in the developing grain endosperm using light microscopy and scanning electron microscopy (SEM) (Figures 2B–C). A-granules were initiated at 10 DPA in both groups, while B-granules occurred at 10 DPA in the high-N treatment group, sooner than in the control group (Figure 2C). Both types of starch granules increased gradually over time under both treatments; however, the high-N treatment group had more and larger A- and B-granules and protein bodies compared with the control group, and this was confirmed by both SEM and light microscopy. These results showed that high-N fertilization significantly promoted starch granule formation and protein body development during grain development.

Gluten macropolymer content detected by SE-HPLC showed that the high nitrogen group (13298.17 mAU) were significantly higher than the control group (11882.9 mAU), increasing by approximately 11% (*p* < 0.05) (Figure S3). Meanwhile, most of the quality parameters, including basic quality properties, farinograph and RVA parameters and breadmaking quality were significantly improved under high-N treatment (Zhen et al., 2016).

### Phosphoproteomic characterization in response to high-N fertilizer

For further elucidation of how high-N fertilizer application influences grain yield and bread quality at the phosphoproteome level, comparative phosphoproteomic analysis of developing grains was carried out from 15 and 25 DPA under high and normal N conditions, respectively. In total, 2047 (83%), 265 (11%), and 158 (6%) identified phosphorylation sites belonged to class I (*p* ≥ 0.75), class II (0.75 > *p* ≥0.5), and class III (*p* < 0.5), respectively (Tables S3A–C; Figure S4A). To improve accuracy, only phosphorylation sites belong to class I were used for subsequent analyses. In total, 2470 phosphosites from 2360 phosphopeptides representing 1372 phosphoproteins were identified in the two groups (Table S1, Figure S4B). Among these 2470 phosphosites, 1969 (80%) were located on serine residues, 455 (18%) on threonine residues, and only 46 (2%) on tyrosine residues (Figure S4C). Most of the phosphoproteins had only one phosphosite, and several had more than 10 sites (Table S2, Figure S4D). We deposited all mass spectrometry data in the ProteomeXchange Consortium via the Proteomics Identifications partner repository with the dataset identifier PXD004128.

**Figure 3:**
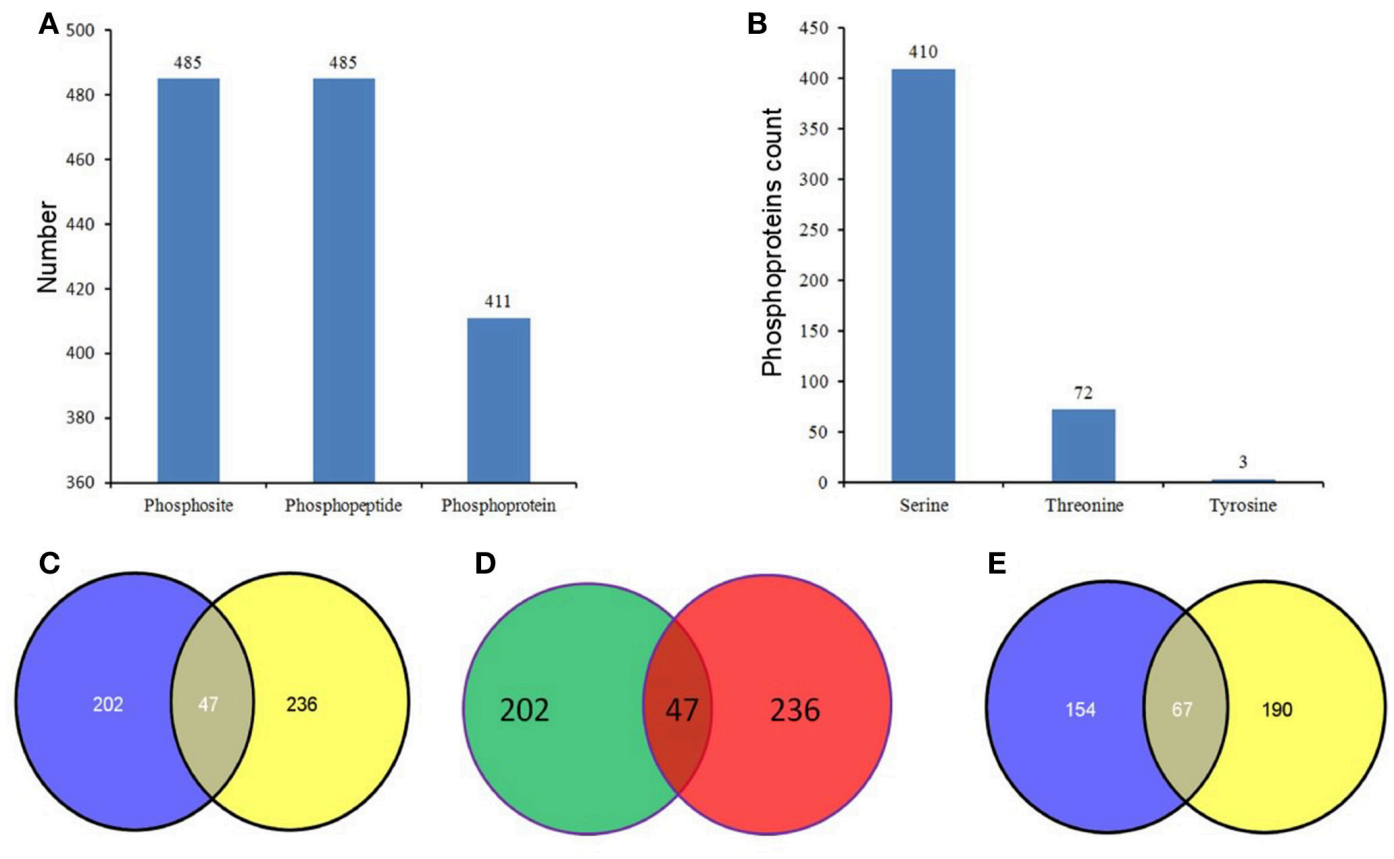
**Phosphorylation status in the developing grains of Zhongmai 175 under normal and high N fertilizer. (A)** The number of phosphosites, unique phosphopeptides, and phosphoproteins with SCPL in CH and treated groups. **(B)** The distribution of SCPL phosphosites on Ser, Thr and Tyr. **(C)** The common phosphosites of the SCPL in CK and high N treated groups. **(D)** The common phosphopeptides of the SCPL in CK and high N treated groups. **(E)** The common phosphoproteins of the SCPL in CK and high N treated groups.

Phosphopeptides showing significance by Student's *t*-test (*p* < 0.05), a high phosphorylation site localization probability (≥0.90), significant intensity changes (≥ 2-fold or ≤ 0.5-fold), and high score differences for phosphorylation sites (≥ 7) were considered to exhibit significant changes in phosphorylation level (SCPL). Under high and normal N-fertilizer application, 485 phosphosites corresponding to 485 phosphorylation peptides, representing 411 proteins, were found with SCPL (Table S4, Figure 3). Among these 485 phosphosites, 410 (84%), 72 (15%), and 3 (1%) were detected on serine, threonine and tyrosine residues, respectively (Figure 3B). Comparing the phosphosites between the two groups (control and high N groups), 249 and 283 were apparent at 15 DPA and 25 DPA, respectively (Tables S4A,B). These SCPL proteins shared 47 common sites (Figure 3C) and 47 common peptides (Table 1, Figure 3D). Of the 411 SCPL proteins, 67 were shared by both groups at 15 and 25 DPA (Figure 3E). These proteins included eukaryotic translation initiation factor isoform 4G-2, zinc finger protein and serine/threonine-protein kinase, among others.

**Table 1:** **The common phosphopeptides that shared by 15 and 25 DPA with SCPL under high N-fertilizer**.

**Protein ID**	**Protein description**	**Localization probablity**	**m/z**	**Phosphorylated peptide**	**Average Intensity (CK-15 DPA)**	**Average Intensity (NT-15 DPA)**	**Average Intensity (CK-25 DPA)**	**Average Intensity (NT-25 DPA)**	**Fold change (CK/NT 15 DPA)**	**Fold change (CK/NT 25 DPA)**
gi|75102610	Eukaryotic translation initiation factor isoform 4G-2	1	496.92	_VSS(ph)INKPSPINPR_	116.97 ± 3.43	38.15 ± 2.15	8.1 ± 1.15	53.1 ± 1.22	3.07	0.15
gi|110729318	ADP-glucose pyrophosphorylase large subunit	1	519.75	_ACIS(ph)PVRR_	54.15 ± 6.25	26.68 ± 1.43	50.35 ± 6.53	19.53 ± 4.47	2.03	2.58
gi|219733995	unnamed protein product	1	412.20	_DRLS(ph)GPSPLGR_	77.12 ± 5.01	21.78 ± 4.24	0.00	20.06 ± 0.93	3.54	—–
gi|475560550	1-phosphatidylinositol-3-phosphate 5-kinase	1	152.37	_NSMS(ph)QHLHDSEIK_	43.63 ± 4.77	21.11 ± 6.65	6.02 ± 0.65	39.80 ± 1.33	2.07	0.15
gi|257135793	WALI7	1	747.29	_VDS(ph)EGVMCGANFK_	6.92 ± 0.05	0.00	9.75 ± 0.53	0.00	—–	—–
gi|359951774	MYB-related protein	1	420.87	_RKS(ph)IHDITNP_	25.29 ± 7.7	5.77 ± 9.1	11.57 ± 5.7	63.78 ± 2.1	4.38	0.18
i|473732123	hypothetical protein TRIUR3_11652	1	648.24	_HVDAS(ph)DDDVDK_	1.29 ± 0.2	0.00	2.81 ± 0.12	0.00	—–	—–
gi|473776495	hypothetical protein TRIUR3_22621	1	672.87	_VLSGTVHILT(ph)PK_	5.6 ± 1.7	13.97 ± 1.42	17.72 ± 3.6	6.8 ± 0.56	0.40	2.61
gi|473817519	AP-1 complex subunit mu-1	1	1077.01	_MEVTQRPPMAVT(ph)NAVSWR_	76.19 ± 1.49	32 ± 2.1	6.2 ± 0.36	21.56 ± 0.63	2.38	0.29
gi|473860118	Regulator of nonsense transcripts 3A	1	404.52	_HLHGS(ph)GPIGEK_	23.47 ± 4.27	6.96 ± 0.42	15.32 ± 1.66	0.00	3.37	—–
gi|473877975	hypothetical protein TRIUR3_27857	1	479.69232	_MLMGDS(ph)PK_	3.54 ± 0.39	0.00	3.69 ± 0.3	0.00	—–	—–
gi|473888536	Cold shock protein CS66	1	851.38324	_HTGAIGT(ph)GLHGADTGEK_	34.11 ± 5.88	7.084 ± 5.12	0.00	11.7 ± 1.65	4.82	—–
gi|473900485	hypothetical protein TRIUR3_31198	1	410.51062	_(ac)S(ph)LMPWFGGGR_	38.32 ± 4.00	11.45 ± 1.73	0.00	25.62 ± 1.87	3.35	—–
gi|473951101	hypothetical protein TRIUR3_33086	1	842.84088	_DRS(ph)MATTQTHDADR_	0.00	5.63 ± 0.92	0.00	21.19 ± 4.85	—–	—–
gi|474036294	DEAD-box ATP-dependent RNA helicase 8	1	624.28702	_FRT(ph)EDVTATK_	116.22 ± 2.45	23.89 ± 0.44	0.00	51.06 ± 3.25	4.86	—–
gi|474036345	Chaperone protein dnaJ 10	1	663.26	_LNSGEGEGS(ph)HMK_	47.54 ± 2.96	16.56 ± 3.3	13.57 ± 1.31	35.72 ± 1.6	2.87	0.38
gi|474036630	Leucine aminopeptidase 2	0.99	816.41	_GHTS(ph)AAASAVPALGLTK_	30.95 ± 4.2	7.48 ± 0.64	0.00	38.65 ± 3.23	4.14	—–
gi|474066052	Zinc finger protein ZPR1	1	400.86768	_IPHGS(ph)VGAVAGR_	110.15 ± 14.75	54.85 ± 8.93	15.18 ± 1.3	99.68 ± 2.8	2.01	0.15
gi|474108764	Serine/threonine-protein kinase fray2	1	727.8556	_KSAS(ph)VGDWIVNAK_	6.19 ± 0.95	0.00	8.54 ± 1.54	0.00	—–	—–
gi|474117335	hypothetical protein TRIUR3_26440	1	516.86	_GRDS(ph)DDDDFRDR_	10.19 ± 0.87	3.35 ± 0.75	3.19 ± 0.26	0.00	3.04	—–
gi|474201675	Serine/arginine-rich splicing factor 7	1	462.72	_IGAGGLGS(ph)GR_	85.83 ± 15.45	31.37 ± 2.44	12.82 ± 0.53	38.63 ± 8.8	2.74	0.33
gi|474252981	hypothetical protein TRIUR3_19373	1	809.36	_RPHT(ph)PIGDEVECK_	9.40 ± 1.63	4.15 ± 1.19	4.43 ± 2.3	0.00	2.26	—–
gi|474307163	putative DNA repair protein RAD23	1	977.97	_APASQSQPAT(ph)PPAPVASAAR_	34.95 ± 2.33	16.60 ± 4.44	21.21 ± 2.44	54.99 ± 12.7	2.11	0.39
gi|474311310	Protein IQ-DOMAIN 32	1	698.84	_VVPLDSDIS(ph)FPK_	56.52 ± 4.13	28.05 ± 2.36	3.79 ± 0.58	0.00	2.01	—–
gi|474326915	Villin-4	1	544.93	_GRS(ph)PAFAALTSAFEK_	3.96 ± 0.28	11.31 ± 0.55	27.02 ± 2.95	5.45 ± 1.83	0.35	4.95
gi|474388024	Elongation factor 1-alpha	1	708.31	_GFVASNS(ph)KDDPAK_	12.31 ± 1.36	2.89 ± 0.27	12.81 ± 3.15	0.00	4.26	—–
gi|474404680	hypothetical protein TRIUR3_12192	1	586.26	_GFFFHPS(ph)PR_	0.00	2.58 ± 0.41	2.39 ± 0.47	0.00	—–	—–
gi|474409971	hypothetical protein TRIUR3_19251	1	880.38	_MLPTGAEEHHPAS(ph)FR_	18.34 ± 1.59	39.52 ± 4.15	14.73 ± 2.35	29.53 ± 4.94	0.46	0.50
gi|474424964	hypothetical protein TRIUR3_15284	1	669.83	_LRGS(ph)QEGLDKR_	65.95 ± 5.61	20.39 ± 1.63	0.00	17.63 ± 1.25	3.23	—–
gi|475542642	Histone-lysine N-methyltransferase	0.98	618.29	_VTPSTS(ph)AVHEK_	5.97 ± 0.98	2.73 ± 0.42	0.00	7.75 ± 1.79	2.19	—–
gi|475552974	Auxin-induced protein	0.98	465.97955	_GVTQQVSAESS(ph)LKGHPR_	13.74 ± 1.22	4.35 ± 1.26	5.09 ± 1.92	35.08 ± 9.84	3.16	0.15
gi|475555787	Zinc finger CCCH domain-containing protein 22	1	518.24	_FS(ph)PGHGAIGL_	4.87 ± 0.58	11.32 ± 1.06	7.43 ± 1.48	17.43 ± 2.28	0.43	0.43
gi|475560550	1-phosphatidylinositol-3-phosphate 5-kinase	1	803.34	_NSMS(ph)QHLHDSEIK_	43.63 ± 4.78	21.11 ± 6.65	6.02 ± 0.65	39.8 ± 1.33	2.07	0.15
gi|475564505	hypothetical protein F775_52380	1	583.81	_S(ph)VGTLIQLQK_	6.81 ± 0.28	3.18 ± 0.25	2.07 ± 0.6	0.00	2.15	—–
gi|475574469	hypothetical protein F775_52479	1	812.40	_SHS(ph)ISNDLHAVQPDPVAADILR_	97.18 ± 6.37	42.28 ± 5.69	12.16 ± 2.14	42.87 ± 4.57	2.30	0.28
gi|475574533	Receptor-like protein kinase FERONIA	1	529.74	_HFS(ph)FAEIK_	0.00	3.42 ± 0.62	4.12 ± 0.03	0.00	—–	—–
gi|475575507	Pumilio-like protein	1	597.80	_GGDGLIGLS(ph)LGR_	29.39 ± 2.67	11.85 ± 1.57	2.21 ± 0.54	0.00	2.48	—–
gi|475591398	hypothetical protein F775_30901	1	484.25	_GALGGS(ph)GLKK_	140.83 ± 1.38	51.8 ± 1.69	21.39 ± 3	126.27 ± 1.41	2.72	0.17
gi|475594692	Serine/threonine-protein kinase CTR1	1	1029.4757	_GKPLDWVS(ph)GQPVTDEHGR_	123.53 ± 1.83	44.48 ± 3.35	0.00	120.97 ± 1.5	2.78	—–
gi|475595539	Coatomer subunit delta-1	1	389.86757	_RAS(ph)ELDKIR_	17.77 ± 3.3	6.35 ± 1.51	0.00	22.78 ± 1.02	2.80	—–
gi|475605268	mRNA decapping complex subunit 2	1	902.9533	_NPS(ph)GVFVPVENPVITR_	152.11 ± 14.9	63.48 ± 0.61	10.16 ± 0.8	66.07 ± 7.72	2.40	0.15
gi|475605485	Inositol hexakisphosphate and diphosphoinositol-pentakisphosphate kinase 1	1	879.90655	_QGS(ph)GIIGTFGQSEELR_	5.35 ± 0.36	0.00	1.28 ± 0.36	0.00	—–	—–
gi|475605563	Putative respiratory burst oxidase-H-like protein	1	477.23516	_VTS(ph)FRVPDMGLK_	9.27 ± 0.6	33.92 ± 0.62	76.22 ± 13.2	32.89 ± 2.5	0.27	2.32
gi|475609742	Adenylyltransferase and sulfurtransferase MOCS3 2	1	602.94633	_PGLAHADS(ph)LPADMIYR_	29.28 ± 2.82	13.95 ± 3.11	14.48 ± 2.27	0.00	2.10	—–
gi|475612820	TOM1-like protein 2	1	754.66808	_VPDDFVNPTAPANMSAPS(ph)HSK_	118.25 ± 7.55	34.49 ± 2.45	7.14 ± 2.26	24.61 ± 3.43	3.43	0.29
gi|475617284	hypothetical protein F775_31415	1	598.73697	_AM(ox)QGWHS(ph)QR_	34.86 ± 6.13	13.91 ± 4.19	0.00	23.69 ± 5.24	2.51	—–
gi|475626660	Ankyrin repeat and zinc finger domain-containing protein 1	1	532.74786	_VAHS(ph)AGSSLR_	6.18 ± 0.47	0.00	0.00	12.49 ± 2.7	—–	—–

*Protein ID: The serial number of the protein identified by MaxQuant in the NCBI database; Protein description: The name of the protein in the NCBI database; Localization probability: The localization probabilities of each phosphorylated site; m/z: mass to charge ratio; Phosphorylated peptide: The identified phosphorylated peptides; Average Intensity: The average expression quantities of each phosphorylated site in three biological repetitions; Fold change: The fold changes of CK group compared with high nitrogen group at 15 and 25 DPA, repectively. Red color means that when CK compare with high nitrogen group, the expression is up-regulate, green color means down-regulate, “−” means the expression quantity of the group is 0*.

These SCPL proteins were used to perform a principal component analysis (PCA), to identify the main sources of variation and reveal hidden structures present in the dataset. As shown in Figure S5A, two sets of samples were grouped differently according to two grain developmental periods. The three biological replicates were highly consistent, and the phosphorylation levels at 15 DPA and 25 DPA showed significant differences via distinct grouping in the PCA plot. These results indicate that variation in developmental stage in phosphorylation level is greater than that in the high-N treatment. Some potential outliers were identified (Figure S5B); these proteins may have more important roles at the phosphorylation level in regulating the plant's response to its N nutrition state. Among these potential outliers, some phosphoproteins, such as sucrose synthase 1, transcription factor VIP1, glyceraldehyde-3-phosphate dehydrogenase and 40S ribosomal protein S20, were closely related to energy metabolism and starch/protein development.

**Figure 4:**
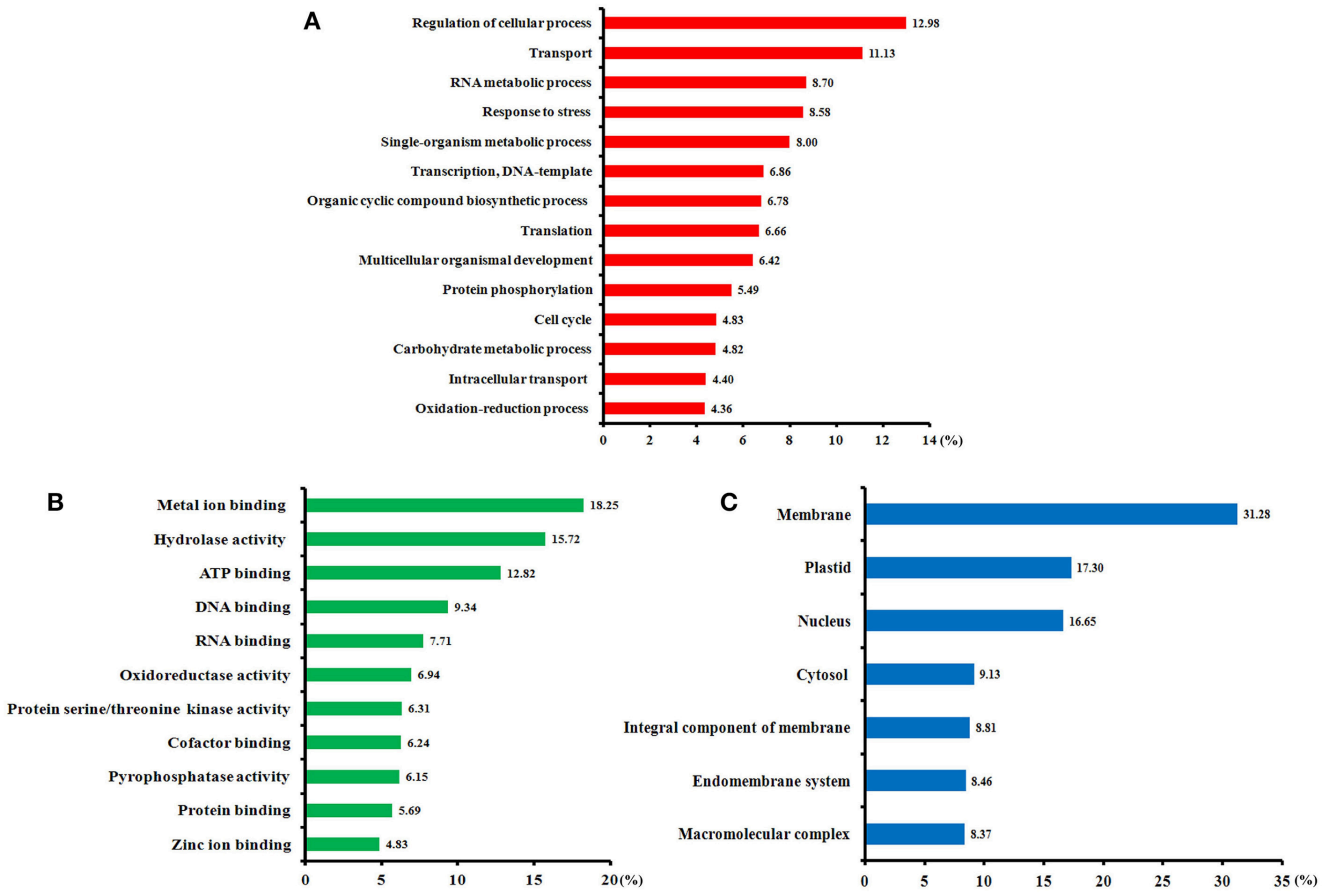
**Functional categories of the phosphorylated proteins identified in the developing grains of Zhongmai 175 under high N fertilizer conditions based on Blast2GO annotation. (A)** Biological processes, **(B)** molecular functions, and **(C)** cellular components.

Of all 411 identified SCPL proteins, 349 (85%) were annotated using Blast2GO software (Table S5). Gene ontology (GO) annotation according to three categories, biological processes, molecular functions, and cellular components, is shown in Figure 4. From a biological process perspective (Figure 4A), most of the identified phosphoproteins participate in the regulation of cellular processes, transport, RNA metabolism, stress response, transcription, and translation. From a molecular function perspective (Figure 4B), the metal ion binding, hydrolase activity, ATP binding, and DNA binding terms were significantly enriched. In the cellular component category (Figure 4C), the membrane, plastid, nucleus, and cytosol terms were significantly enrichment. Our GO annotation results indicate that high-N fertilization affected various metabolic processes during wheat grain development.

### Phosphorylation motif and conservation analysis of the significantly altered phosphopeptides

**Figure 5:**
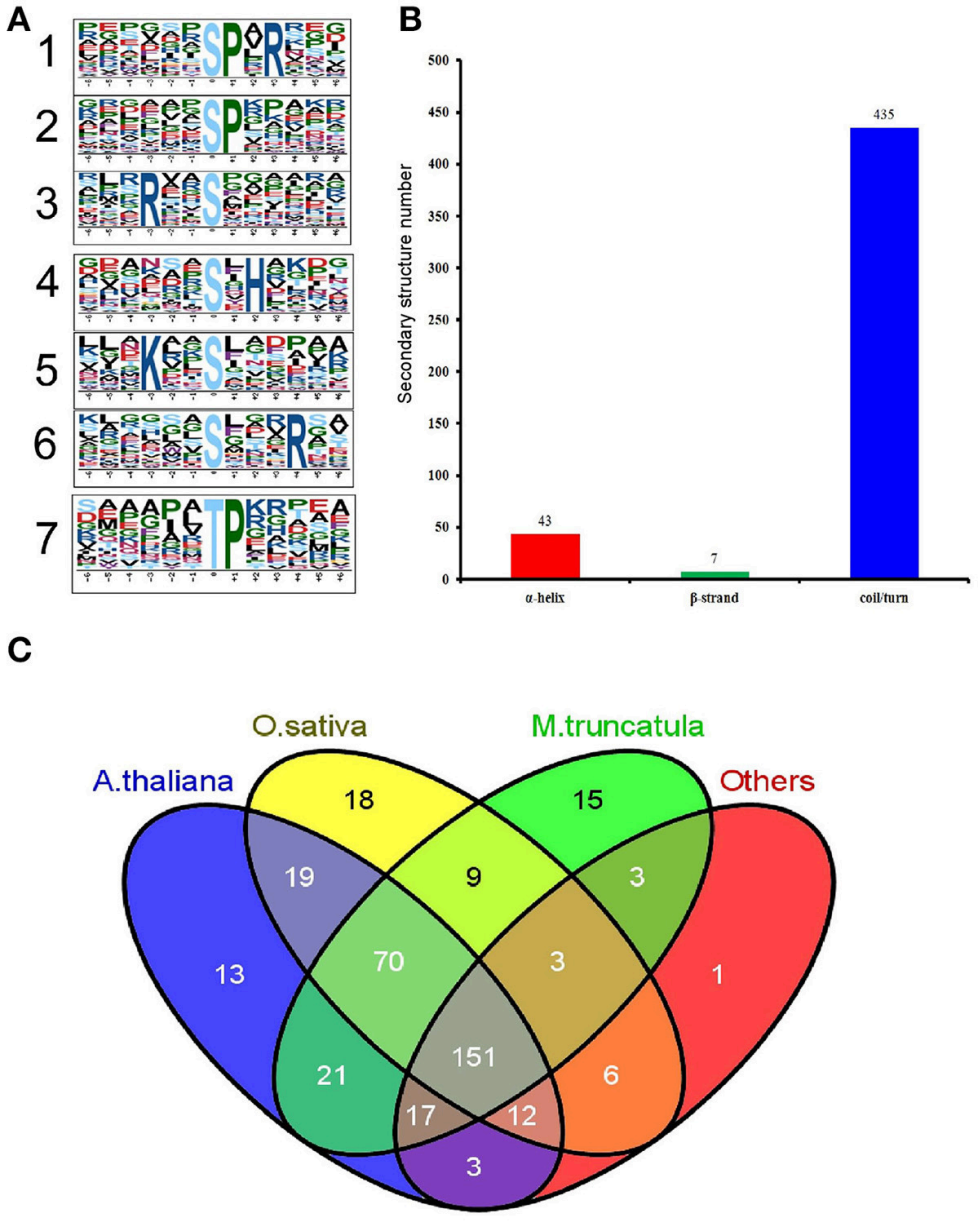
**Properties of the significantly changed phosphopeptides from high N fertilizer. (A)** Motif analysis of the amino acids surrounding the identified phosphorylated residues by Motif-X. The height of each letter corresponds to the frequency of that amino acid residue in that position. The central S/T refers to the Ser/Thr amino acids residues. **(B)** The number of secondary structures (α-helix, β-strand, coil) of phosphosites in the proteins with SCPL. **(C)** Conservation analysis of the SCPL proteins under different N fertilizer conditions. All the identified SCPL proteins were compared with *Oryza sativa, Arabidopsis thaliana, M. truncatula*, and others.

Motif-X software was used to conduct a motif analysis of the significantly altered phosphopeptides, as shown in Figure 5A. Seven phosphopeptide motifs were enriched, including six serine motifs (-SP-R, -SP-, -R–S-, -S-H-, -K–S-, and -S—R-) and one threonine motif (-TP-) (Tables S6, S7). Two distinct types of residues were located upstream or downstream of the phosphorylated residues: positive charged residues including histidine (H) or lysine (K) and residues with a nonpolar R group such as proline (P). This may result in a negative charged phosphate group that can combine easily with positive charged groups. The most common combination, represented by 72 (28%) of the enriched motifs, was -R–S- (Table S7), a basic motif that is a potential substrate for calmodulin kinase-II, protein kinase A and protein kinase C. According to a previous study (Durek et al., 2010), -SP- is a proline-directed motif recognized by mitogen-activated protein kinases (MAPKs), cyclin-dependent kinases (CDKs), and CDK-like kinases. The -S-H- motif, identified for the first time in our study, will be recognized by these protein kinases and subsequently phosphorylated.

We used the NetSurfP algorithm to predict the secondary structures of these phosphorylated sites (Figure 5B). The results showed that coil/turn, α-helix, and β-strand structures accounted for 90, 9, and 1%, respectively. Most of the phosphorylated sites were located in the loose area, to allow this large phosphorylation group to combine with the proteins more easily.

We performed a Basic Local Alignment Search Tool (BLAST) search of phosphoprotein databases using the sequences of the identified proteins that showed significant differences and estimated the degree of conservation among different species: *T*. *aestivum, Medicago truncatula, Oryza sativa, A. thaliana*, and other plant species (Figure 5C). These databases were constructed using data from the Plant Protein Phosphorylation DataBase, Medicago-Omics Repository, and PhosPhAt 4.0. The following parameters were set: score ≥ 80, *E* < 1 × 10^−10^, and identity ≥ 30%. The results showed that 361 (88%) phosphoproteins had orthologous proteins in four other organisms, indicating high conservation of the wheat phosphoproteome. Furthermore, 151 (37%) phosphoproteins were shared among all these several species; 306 (74%) wheat phosphoproteins were shared with *A. thaliana* (average score 557, average identity 58%) (Table S8); 288 (70%) were shared with rice (average score 777, average identity 67%); and 290 (71%) were shared with *M. truncatula* (average score 534, average identity 104%). These results indicate that *T. aestivum* phosphoproteins had the highest similarity to *O. sativa* phosphoproteins, followed by *A. thaliana*.

### Screening of significant phosphorylated peptides and proteins in response to high-N fertilizer and their molecular structural characteristics

**Figure 6:**
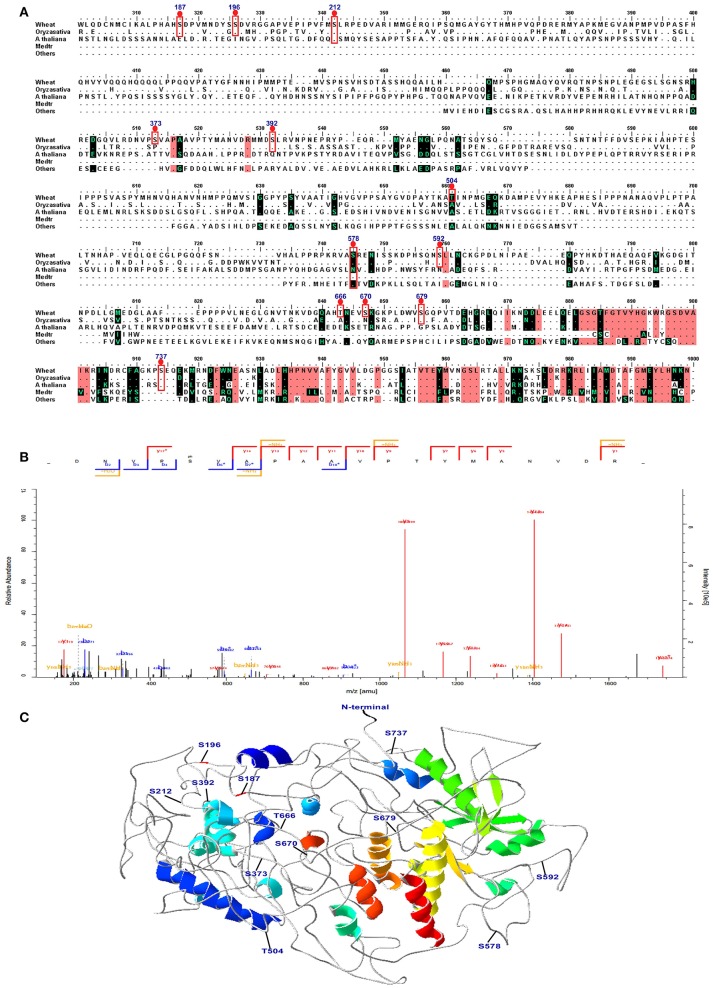
**Sequence alignment, spectra map for tandem mass spectrometry (MS/MS) and three-dimensional structure of serine/threonine-protein kinase CTR1. (A)** The sequence alignment of CTR1 in different species. The phosphosites are marked with red box. **(B)** The representative image of the phosphopeptide (DNVPS_(ph)_VAPAAVPTYMANVDR) in CTR1. **(C)** The 3D structure of CTR1. The phosphosites are highlighted in the structure.

Combining the conservation analysis, PCA analysis and the common peptides of the 15 DPA and 25 DPA groups, several proteins with significant changes in their phosphorylation level were identified under high-N fertilizer treatment, including DEAD-box ATP-dependent RNA helicase 8, chaperone protein dnaJ 10, serine/threonine-protein kinase fray 2, serine/arginine-rich splicing factor 7, receptor-like protein kinase feronia, respiratory burst oxidase-H-like protein, and eukaryotic translation initiation factor isoform 4G-2. The peptide GKPLDWVS(ph)GQPVTDEHGR, which belongs to the serine/threonine protein kinase CTR1 and may play vital roles in grain development, was significantly altered. According to the PCA analysis, site 1660 belongs to the serine/threonine-protein kinase CTR1, and was separate from most of the proteins (Figure S5B). Thus, we further analyzed the three-dimensional (3D) structure and performed a sequence alignment of serine/threonine protein kinase CTR1 with those of other plant species, as shown in Figure 6. In the protein serine/threonine protein kinase CTR1, 12 phosphosites were identified on serine/threonine residues, which are marked on the sequence alignment in Figure 6A. The representative spectrum of the phosphopeptide DNVPS_(ph)_VAPAA VPTYMANVDR in this protein is shown in Figure 6B, and its 3D structure is shown in Figure 6C. These 12 phosphorylated sites were found to be located in coil/turn regions, which may allow the phosphate group to interact more easily with the loose area. Furthermore, we also predicted the 3D structures of receptor-like protein kinase (gi/475574533), eukaryotic translation initiation factor isoform 4G-2 (gi/75102610), chaperone protein dnaJ 10 (gi/474536345), and serine/arginine-rich splicing factor 7 (gi/474201675), as shown in Figure S6. All results indicated that the phosphorylation sites are located mostly in coil areas. This was also verified by the prediction of secondary structures as shown in Table S9. Based on the alignment for this protein, most of the phosphorylated peptides in wheat were found to be similar to those in Bd21, indicating that *T. aestivum* has closer relationships to Bd21 than to other species (Figure 6A).

### Protein-protein interaction analysis of phosphoproteins

These identified SCPL proteins were analyzed by STRING to build PPI networks (Figure S7, Tables S10, S11). To improve the reliability of PPI analysis, the confidence score was set at the highest level (≥ 0.9). According to the functional categories, several groups were identified: translation/transcription ranked first, followed by kinase/phosphatase, energy metabolism, transportation, stress response, and cell division/development (Table S10).

**Figure 7:**
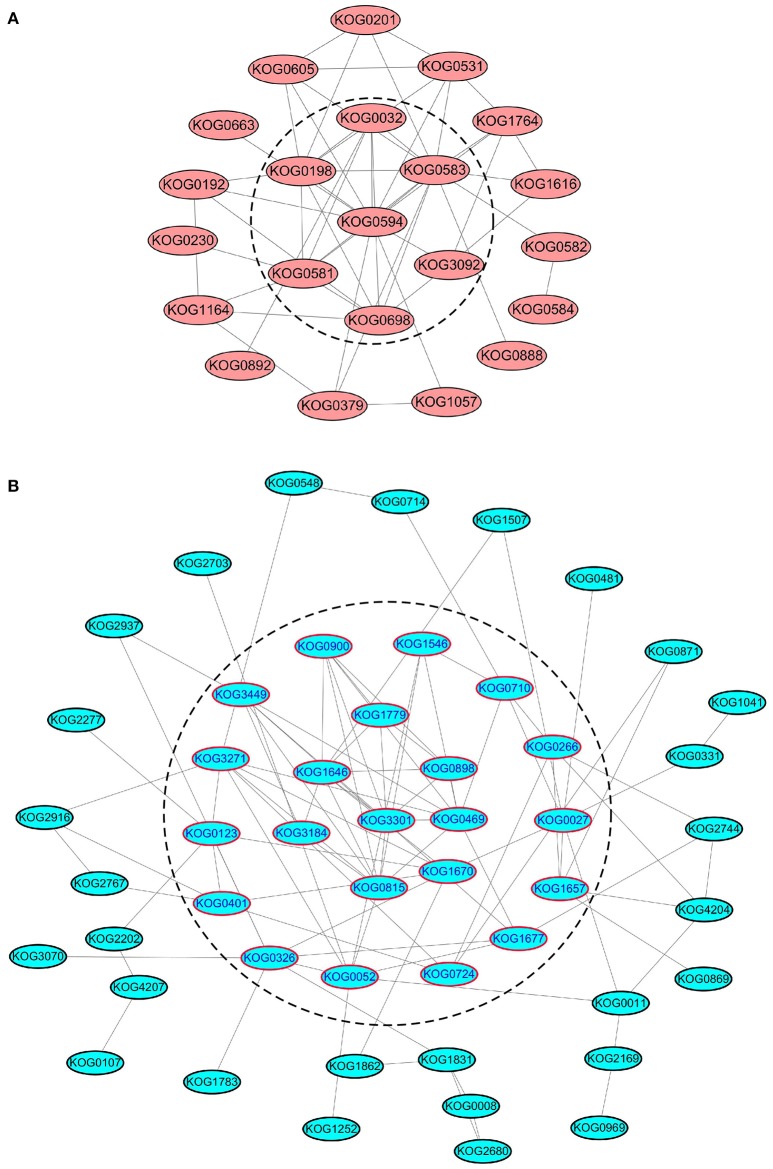
**PPI analysis of SCPL phosphoproteins of kinases/phosphatases and translation/transcription factors. (A)** The PPI analysis of all the kinases/phosphateases. **(B)** The PPI analysis of all the translation/transcription factors. The proteins in the dash line means that they are located at the central place of the PPI network.

We further analyzed the PPI networks of all kinases/phosphatases and translation/transcription factors separately (Figure 7). Apparently, calmodulin-dependent protein kinase (CDPK), serine/threonine-protein kinase sepA, SNF1-related protein kinase, CDK F-1, MAPK kinase 1, casein kinase II, and serine/threonine protein phosphatase are closely related (Figure 7A). These kinases/phosphatases are closely related to energy metabolism and translation/transcription (Figure S7), indicating their important roles in protein substrate phosphorylation/dephosphorylation under different levels of N fertilization. Several ribosomal proteins were intimately interconnected via elongation factors and eukaryotic translation initiation factors (Figure 7B), suggesting that these proteins are sensitive to the addition of N fertilizer and are involved in protein synthesis.

### Verification of phosphorylated proteins by Pro-Q diamond staining and western blotting

**Figure 8:**
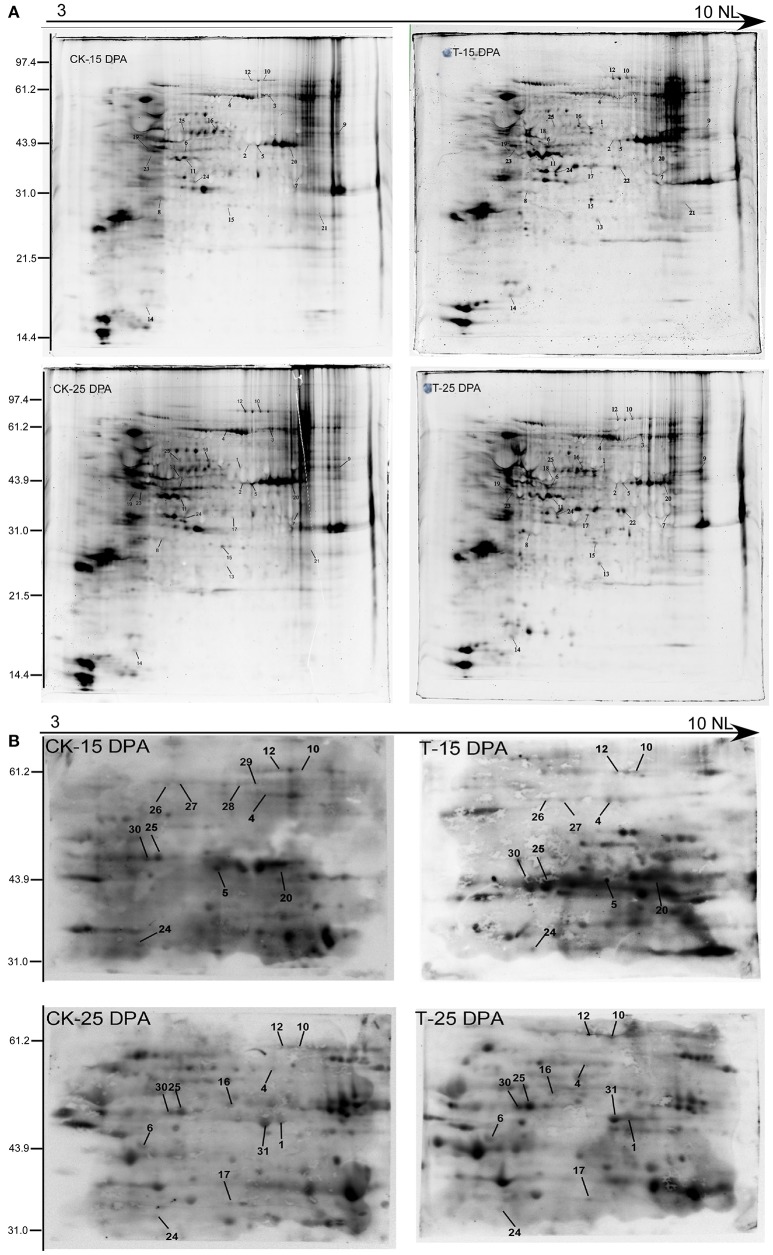
**The verification of phosphoproteins with Pro-Q diamond staining (A)** and Western blotting analysis **(B)** of the protein samples from 15 and 25 DPA under high and normal N conditions.

To further verify the reliability of our phosphoproteomic dataset, two-dimensional electrophoresis (2-DE) and Pro-Q Diamond staining (Invitrogen, USA) were performed as shown in Figure 8A, resulting in hundreds of fluorochrome-stained protein spots. The Pro-Q diamond phosphoprotein stain specifically binds to the phosphate moieties of phosphoproteins, disregarding phosphoamino acids, thereby allowing direct detection of phosphoproteins (Silva-Sanchez et al., 2015). Thus, 2-DE can be employed as a high-resolution technique that takes advantage of the pI and molecular weight changes associated with PTMs. Coomassie brilliant blue (CBB) staining of 2-D gels was performed to visualize total proteins, and some phosphoproteins were identified by MALDI-TOF/TOF. In total, 25 proteins were identified and matched well to those from the phosphoproteomic dataset, such as phosphoglucomutase, fructose 1-,6-biphosphate aldolase, glyceraldehyde-3-phosphate dehydrogenase, sucrose synthase 2, and several hypothetical proteins, shown in Table S12.

Western blotting using an antibody recognizing phosphorylated proteins was performed for further detection of the phosphoproteins on the 2-DE gels in the 15 and 25 DPA groups (Figure 8B). Sixteen phosphorylated proteins were identified that matched with those in our phosphoproteomic dataset, such as fructose 1-,6-biphosphate aldolase, elongation factor 1, and sucrose synthase 2 (Table S13).

## Discussion

In this study, we performed a quantitative phosphoproteomic analysis under high-N fertilization conditions and identified many phosphoproteins related to high-N response and grain development. We discuss the potential functions of central phosphoproteins involved in different metabolic processes during grain development, particularly for grain starch and protein synthesis, which are closely related to yield and bread quality formation.

### Protein kinases (phosphatases) participate in signal perception and transduction

Through the phosphorylation/dephosphorylation cascade controlled by kinases or phosphatases, the external changes in available N supply can be recognized; this results in N uptake and transduction into intracellular spaces, where N-responsive proteins will be directly or indirectly phosphorylated or dephosphorylated to adapt to external nutrient changes. In this study, 52 kinases or phosphatases were significantly altered at the phosphorylation level under high-N treatment (Tables S4A,B).

For N-related processes, CBL-interacting protein kinases CIPK8 and CIPK23, regulate the expression of nitrate-responsive genes, including nitrate transporter-encoding genes and genes required for N assimilation, and affect signaling activity when N availability drops (Ho et al., 2009; Hu et al., 2009). CIPK23 interacts with CHL1 as a nitrate transporter in the plasma membrane important for CHL1 phosphorylation in response to low nitrate concentrations, dephosphorylated CHL1 will lead to high level nitrate response, and CIPK23 also serves as a negative regulator of the high-affinity nitrate response (Ho et al., 2009). Interestingly, we found nine phosphorylation sites in CIPK23 (Table S1), which exhibited a significant decrease (−3.3-fold) in their level of phosphorylation at 15 DPA, thus the phosphorylation level of CHL1 at T-101 site will also reduce, which will tend it to a low-affinity nitrate transporter in response to high level nitrate concentration.

Recently, Quan et al. (2016) identified 85 kinases that responded to N fertilization through barley transcriptomic profiling, including serine threonine-protein kinase (STK), leucine-rich repeat receptor-like kinase, and calmodulin-binding receptor-like cytoplasmic kinase. Similarly, one putative STK, similar to the yeast SNF1 protein kinase, was found to be responsive to nitrate in Arabidopsis through microarray and RNA gel blot analyses (Wang et al., 2000). Many kinase families in *Arabidopsis* have been found to be phosphorylated upon nitrate or ammonium resupply after N starvation, such as receptor-like kinases (RLKs), Snf1-related protein kinases (SnRKs), BR signaling kinases, MAPK pathway members, casein kinase and 1-phosphatidylinositol-4-phosphate 5-kinase (PI kinase) (Engelsberger and Schulze, 2012).

In the present study, RLKs, SnRKs, MAPKs, and casein kinases were also phosphorylated. STK CTR1 showed significantly different phosphorylation levels at both 15 and 25 DPA (Table 1). This protein is well conserved in these several species (Figure 5C). The sequence alignment and 3D structure indicated that all phosphorylated sites are localized in the loop region (Figure 6C). SnRKs are central players linking stress responses and metabolic signaling (Fragoso et al., 2009). In addition, NR and SPS are the substrates of SnRKs, and SnRK1 protein kinases are global regulators of carbon metabolism in plants (Halford and Hardie, 1998). One peptide with an SxxxR motif of this protein at 25 DPA was upregulated 3.64-fold compared with the control group, indicating that protein phosphorylation provides a layer of signaling regulation in response to the changing external N concentration during grain development.

### Regulation of starch biosynthesis via protein phosphorylation

**Figure 9:**
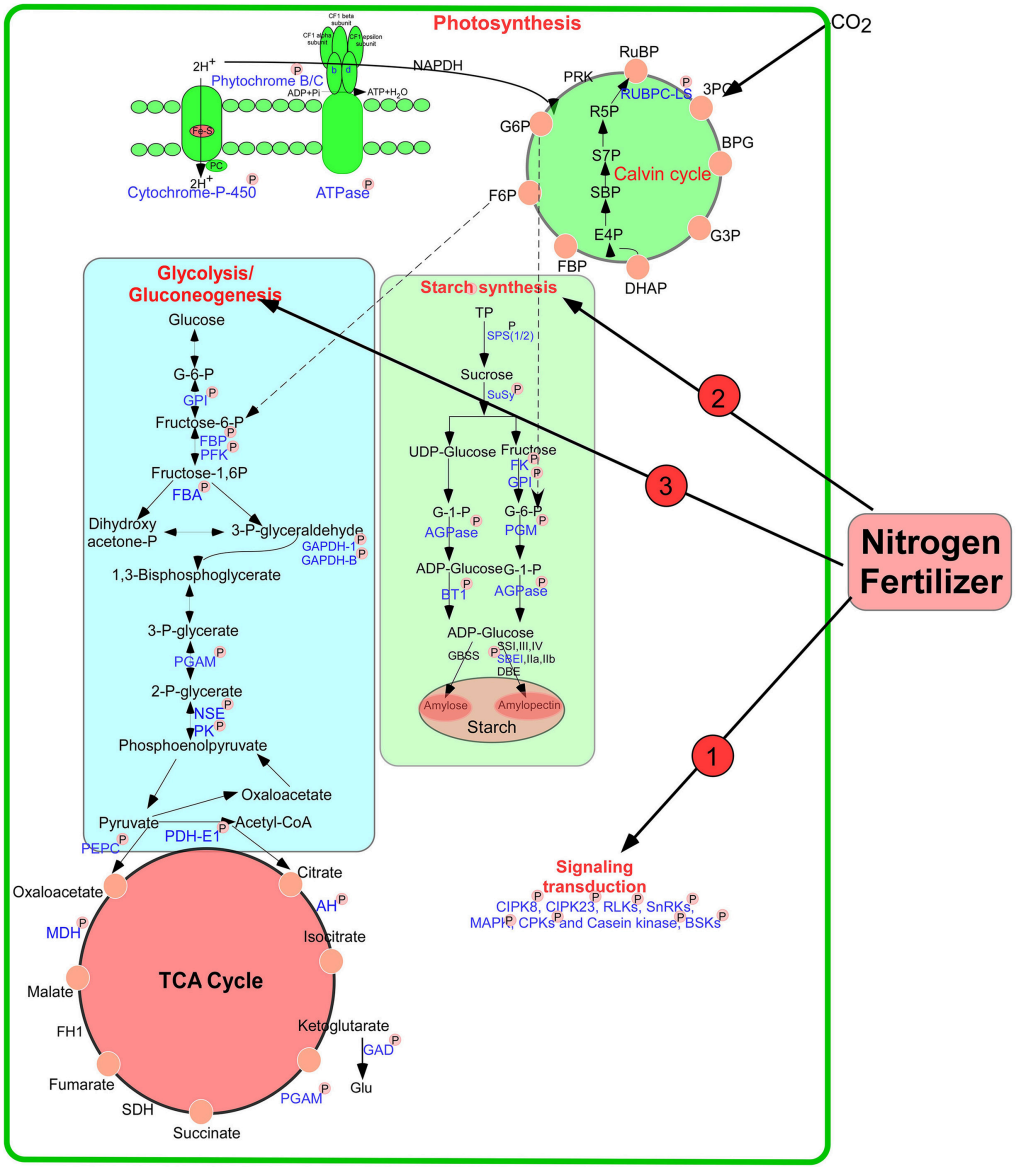
**A putative pathway of protein phosphorylation regulating grain starch and protein synthesis in response to high N fertilizer**. The phosphorylated proteins identified in this study were drawn with blue color and marked with “P.” AGPase: ADP glucose pyrophosphorylase, AH, aconitate hydratase; BT1, ADP-glucose brittle-1 transporter precursor (BT1); DBE, De-branching enzyme; FBA, fructose-bisphosphate aldolase; FBP, fructose-1,6-bisphosphatase; FK, fructokinase; GAPDH, glyceraldehyde-3-phosphate dehydrogenase; GBSS, Granule bind starch synthase; GPI, glucose-6-phosphate isomerase; NSE, enolase; PGAM, 2,3-bisphosphoglycerate-independent phosphoglycerate mutase; PGM, phosphoglucomutase; PK, pyruvate kinase; SPS, sucrose phosphate synthase; SuSy, Sucrose synthase; SBE, starch branching enzyme; SS, Starch synthase; PFK, 6-phosphofructokinase; TP, triose phosphates.

During the early grain developmental stages, photosynthesis provides the raw material triosephosphate for starch biosynthesis (Tschiersch et al., 2010). As shown in Figure 9, a large number of enzymes related to starch synthesis were phosphorylated, including SPS 1/2, SuSy 1/2, starch branching enzyme I (SBEI), glucose-6-phosphate isomerase, AGPase, phosphoglucomutase (PGM), and ADP-glucose brittle-1 transporter precursor (BT1). Many intermediates from the pentose phosphate pathway are released from chloroplasts in the form of triose phosphates for sucrose biosynthesis. SPS is an important control point in this pathway, and its activity is reversibly modulated by phosphorylation in response to light/dark signals (Huber, 2007). Our study found that the activity of this enzyme improved markedly after high-N application (Figure 1G). Particularly, SPS showed SCPL at Ser-285 at 15 DPA (Table S4A). A previous study showed that phosphorylation serves as a mechanism for osmotic stress activation of SPS in spinach leaves (Torose and Huber, 1997). Thus, we speculated that phosphorylation of SPS at Ser-285 could regulate its activity in response to changes in external nutrient availability. In addition, SuSy activity also increased markedly after high-N fertilizer application (Figure 1H), consistent with a previous report (Zou et al., 2008). Phosphorylation at Ser-15 affects amino terminal conformation, which may stimulate the catalytic activity of SuSy (Hardin et al., 2004). However, we found significant upregulation in SuSy1 phosphorylation at Ser-10 at 15 DPA (Table S4A). A previous study demonstrated that phosphorylation improved starch enzyme activity and increased the number of starch granules (Tetlow et al., 2004). Since 15 DPA is a vital period for grain filling, phosphorylation will accelerate starch synthesis.

AGPase, the rate-limiting enzyme in starch biosynthesis, was phosphorylated at Ser-69 under both water-sufficient and water-deficient conditions in a previous study (Zhang et al., 2014a). AGPase is associated with starch biosynthesis and commonly distributed in the plastids of most plant tissues (Huang et al., 2014). We found that phosphosite Ser-19 of AGPase (gi|110729318) showed significant changes at both 15 DPA and 25 DPA. The phosphorylation level of gi|20127139 at Ser-369 was increased markedly at 25 DPA after high-N treatment. Starch content was also improved significantly at a late stage of grain development (Figure 1F). The activity of AGPase after high-N application also significantly increased at 25 DPA (Figure 1I). These results correspond well to a previous report (Xu et al., 2015). AGPase catalyzes glucose-1-phosphate to produce ADP-glucose, which is transported into plastids by a BT1 transporter to participate in starch biosynthesis. Our results demonstrated that phosphorylation regulates the activity of SPS, SuSy, and AGPase to respond to nutrition changes.

Previous studies showed that wheat starch synthesis enzymes such as SSI, SSII-a, SBEI, SBEII-a, and SBEII-b were phosphorylated and participated in protein–protein interactions, and the activities of SBEIIa and SBEIIb in amyloplasts were enhanced by phosphorylation (Tetlow et al., 2004, 2008). Recently, Chen et al. (2016) found that phosphorylation of starch granule-binding proteins occurs during all grain developmental stages and plays a critical role in starch biosynthesis. Coincidentally, we also identified Ser-839 in SBEI, which participates in starch biosynthesis via phosphorylation. Based on these results, we speculate that these phosphorylated proteins enhance the ability of starch biosynthesis to improve wheat grain yield under high N-fertilizer conditions.

### Phosphorylated proteins involved in energy metabolism

Carbon is continuously metabolized in plants and is essential for energy circulation and plant survival. We found that phosphorylated proteins participated in three respiratory pathways: glycolysis, the mitochondrial electron transport chain, and the TCA cycle. These three pathways are essential for energy supply to numerous cellular functions (Fernie et al., 2004).

ATP is consumed to provide energy for protein/amino acid synthesis during nitrate assimilation (Arsova et al., 2012). Interestingly, we found many enzymes/proteins participating in glycolysis and the TCA cycle to be phosphorylated, such as glyceraldehyde-3-phosphate dehydrogenase B, PGM, 6-phosphofructokinase 3 (PFK), enolase, FBA, and pyruvate dehydrogenase E1 component subunit alpha-1 (Figure 9). During glycolysis, the PFK-catalyzed reaction that produces fructose 1, 6-bisphosphate, and ADP from fructose 6-phosphate and ATP is thought to be a major controlling step of the metabolic flux through glycolysis (Hess and Boiteux, 1971). This reaction is regulated by phosphorylation, which appears to promote attachment of the COOH-terminal peptide to the dimer surface via specific ionic interactions (Sprang and Fletterick, 1980) and results in stronger interactions between subunits, leading to different conformation states (Kitajima et al., 1983). Phosphorylation may control the activity of the PFK to regulate metabolic flux in response to the external N availability.

At the beginning of the TCA cycle, the pyruvate dehydrogenase complex (PDC) catalyzes the irreversible conversion of pyruvate, coenzyme A and NAD^+^ into CO_2_, NADH and acetyl-CoA (Patel and Roche, 1990). PDC is the largest and one of the most complex multienzyme systems known (Zhou et al., 2001). In our study, a protein pyruvate dehydrogenase E1 component subunit β was found with SCPL at 25 DPA after high-N fertilizer treatment. Carbon flux through PDC is meticulously controlled by elaborate mechanisms involving post-translational phosphorylation/dephosphorylation and transcriptional controls (Patel and Korotchkina, 2006). Particularly, kinetic and thermodynamic analyses of PDC indicated that phosphorylation blocks access to its active site by imposing a steric and electrostatic barrier for substrate binding and active site coupling with the E2 component (Seifert et al., 2007). The phosphorylation level of this enzyme showed significant downregulation (−2.7-fold) after high-N fertilizer treatment, which promotes the interaction of PDC with E2 components to regulate carbon flux for rapid protein synthesis.

### A putative pathway of protein phosphorylation regulating grain starch and protein synthesis under high-N fertilizer

According to our results and previous reports, we propose putative metabolic protein phosphorylation pathways that regulate grain starch and protein synthesis in response to high-N fertilizer application (Figure 9). External changes in the available N supply were recognized, leading to N uptake and transduction into intracellular spaces, where N-responsive proteins were directly or indirectly phosphorylated or dephosphorylated by kinases or phosphatases, respectively, to allow adaption to the altered nutrient availability. Subsequently, N may be assimilated into amino acids, proteins, and starch, and during these processes, phosphorylation regulates key enzymes involved in starch synthesis and energy metabolism in response to high N concentrations. The wheat plant will be regulated synergistically by phosphorylation to response to the nitrogen changes in the environment.

## Conclusion

High-N fertilizer application led to significant improvements in main agronomic traits, physiological, and biochemical properties, starch, and protein syntheses, and grain yield and quality. Comparative phosphoproteomic analysis at two grain developmental stages (15 and 25 DPA) of Chinese elite bread wheat cultivar Zhongmai 175 under high-N fertilizer was performed to identify 1372 phosphoproteins using label-free quantification. Among them, 411 proteins showed significant phosphorylation changes under high-N fertilizer conditions, associated mainly with signal transduction, starch synthesis and energy metabolism. We propose a putative metabolic pathway involving phosphorylated proteins regulating grain starch and protein synthesis in response to high-N fertilizer. Phosphorylation modification cooperatively regulated the activity of key enzymes or proteins involved in the response to N fertilizer application, accelerated starch, and protein synthesis, and ultimately improved grain yield and bread making quality. Our results from this phosphoproteome-level study provide new insights into the molecular mechanisms of wheat yield and bread quality formation under high-N fertilizer application.

## Author contributions

SZ, XD, and MZ performed all the experiments, data analysis and wrote the paper. GZ, contributed to SE-HPLC and YW help with light microscope. DL and DZ revised the manuscript. YY designed and supervised experiments.

### Conflict of interest statement

The authors declare that the research was conducted in the absence of any commercial or financial relationships that could be construed as a potential conflict of interest.

## Supplementary material

Figure S1 **Schedule of the experiment design of the large-scale phosphoproteomics analysis under normal and high N fertilizer conditions**.

Figure S2 **Phenotype characteristics of wheat plant under high N fertilizer(T) and normal N fertilizer (CK) conditions**.

Figure S3 **Image of SE-HPLC of wheat grains under high N fertilizer condition**.

Figure S4 **Phosphorylation status in the developing grains of Zhongmai 175 under high N fertilizer condition. (A)** Number of phosphosites, unique phosphopeptides, and phosphoproteins identified in CK and treated groups. **(B)** Distribution of phosphosites on Ser, Thr and Tyr. **(C)** Distribution of phosphosites in each phosphoprotein. **(D)** Classification of phosphosites of all the identified sites in two groups.

Figure S5 **PCA analysis of the protein samples and phosphosites in CK and high N fertilizer groups. (A)** PCA of individual protein samples in wheat grains under different N fertilizer conditions. **(B)** PCA of SCPL phosphosites spots in wheat grains under high N fertilizer.

Figure S6 **Sequence alignment and three-dimensional structure of four key phosphorylated proteins (gi475574533, gi75102610, gi474036345 and gi474201675)**. The phosphosites are marked with black box and highlighted in the 3D structure.

Figure S7 **PPI analysis of all the identified phosphoproteins with SCPL under high N fertilizer condition**.

Table S1 **All of the phosphorylated sites and phosphoproteins identified in the developing grains at 15 and 25 DPA from Zhongmai 175 under normal and high N fertilizer conditions**.

Table S2 **The distribution of phosphosites in each protein**.

Table S3A **The classification of the phosphoproteins based on localization probability (*p* ≥ 0.75) of all the identified proteins-Class I**.

Table S3B **Classification of the phosphoproteins based on localization probability (0.5 ≤ *p* < 0.75) of all the identified proteins-Class II**.

Table S3C **The classification of the phosphoproteins based on localization probability (*p* > 0.5) of all the identified proteins-Class III**.

Table S4A **Phosphoproteins with SCPL at 15 DPA under high N fertilizer condition**.

Table S4B **Phosphoproteins with SCPL at 25 DPA under high N fertilizer condition**.

Table S5 **Gene ontology annotation of the phosphoproteins with SCPL under high N condition**.

Table S6 **Motif analysis of phosphoproteins with SCPL under high N condition**.

Table S7 **Detailed information of the motifs extracted from phosphoproteins with SCPL under high N condition**.

Table S8 **Conservation analysis of phosphoproteins with SCPL in wheat with *Medicago truncatula, Oryza sativa, A. thaliana*, Bd-21 and other plant species**.

Table S9 **Possibilities of the secondary structures of the phosphorylated proteins with SCPL under normal and high N fertilizer conditions**.

Table S10 **KOG number and function of the phosphoproteins with SCPL under high N condition**.

Table S11 **PPI networks of SCPL phosphoproteins constructed by STRING**.

Table S12 **Verification of phosphoproteins with Pro-Q staining and MALDI-TOF/TOF**.

Table S13 **Verification of phosphoproteins with Western blotting and MALDI-TOF/TOF**.

## Abbreviations

ACN: acetonitrile
AGPase: ADP glucose pyrophosphorylase
BSKs: BR signaling kinases
BT1: ADP-glucose brittle-1 transporter precursor
CDK: cyclin-dependent kinase
CaMK-II: calmodulin kinase-II
CDPK: calmodulin-dependent protein kinase
CDK: Cyclin-dependent kinase F-1
CK-II: Casein kinase II
CIPK: CBL-interacting protein kinase
CRCK: calmodulin-binding receptor-like cytoplasmic kinase
DPA: days post-anthesis
DTT: dithiothreitol
ERLIC: electrostatic repulsion hydrophilic interaction chromatography
FA: formic acid
FBA: fructose-bisphosphate aldolase
FDR: false discovery rate
IMAC: immobilized metal affinity chromatography
GAPDH: Glyceraldehyde-3-phosphate dehydrogenase
GMP: gluten macropolymers
GO: gene ontology
GPI: glucose-6-phosphate isomerase
HILIC: hydrophilic interaction liquid chromatography
KOG: EuKaryotic Orthologous Groups
LC−MS/MS: liquid chromatography/tandem-mass spectrometry
LRR: leucine-rich repeat receptorlike kinase
MAPKK: Mitogen-activated protein kinase kinase
MOAC: TiO_2_-metal oxide affinity chromatography
MORE: Medicago-Omics Repository
N: Nitrogen
NLA: nitrogen limitation adaptation
NR: nitrate reductase
NSE: enolase
P3DB: Plant Protein Phosphorylation DataBase
PCA: principal component analysis
PDC: pyruvate dehydrogenase complex
PGM: phosphoglucomutase
PFK: phosphofructokinase
PDH: pyruvate dehydrogenase
PKA: protein kinase A
PPI: protein-protein interaction
PolyMAC: polymer based metal ion affinity capture
PTM: post-translational modification
RLKs: receptor-like kinases
SBEI: starch branching enzyme I
SE-HPLC: size exclusion-high performance liquid chromatography
SEM: scanning electron microscopy
SCPL: significant changes in phosphorylation level
STK: serine threonine-protein kinase
SnRKs: Snf1-related protein kinases
SPS: sucrose phosphate synthase
SuSy: sucrose synthses
TFA: trifluoroacetic acid
TFs: transcription factors.
