# Supplementary figures and images for: Comparative Phosphoproteomic Analysis under High-Nitrogen Fertilizer Reveals Central Phosphoproteins Promoting Wheat Grain Starch and Protein Synthesis
Source: Front Plant Sci. 2017 Jan 30;8:67. doi: 10.3389/fpls.2017.00067 (PMC5277015; doi:10.3389/fpls.2017.00067)

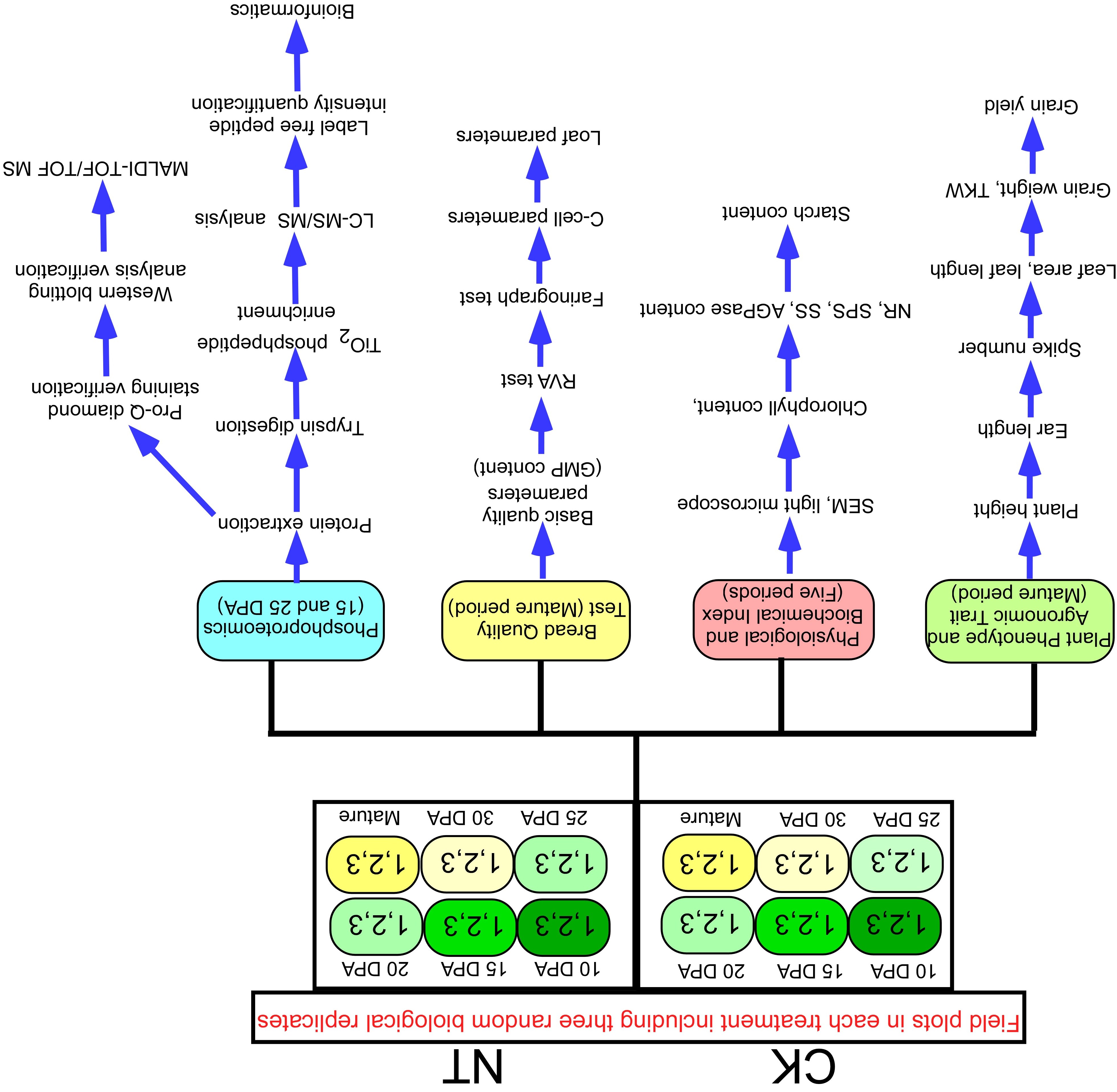

Supplement: Figure S1 — Schedule of the experiment design of the large-scale phosphoproteomics analysis under normal and high N fertilizer conditions. [file Image1.JPEG]

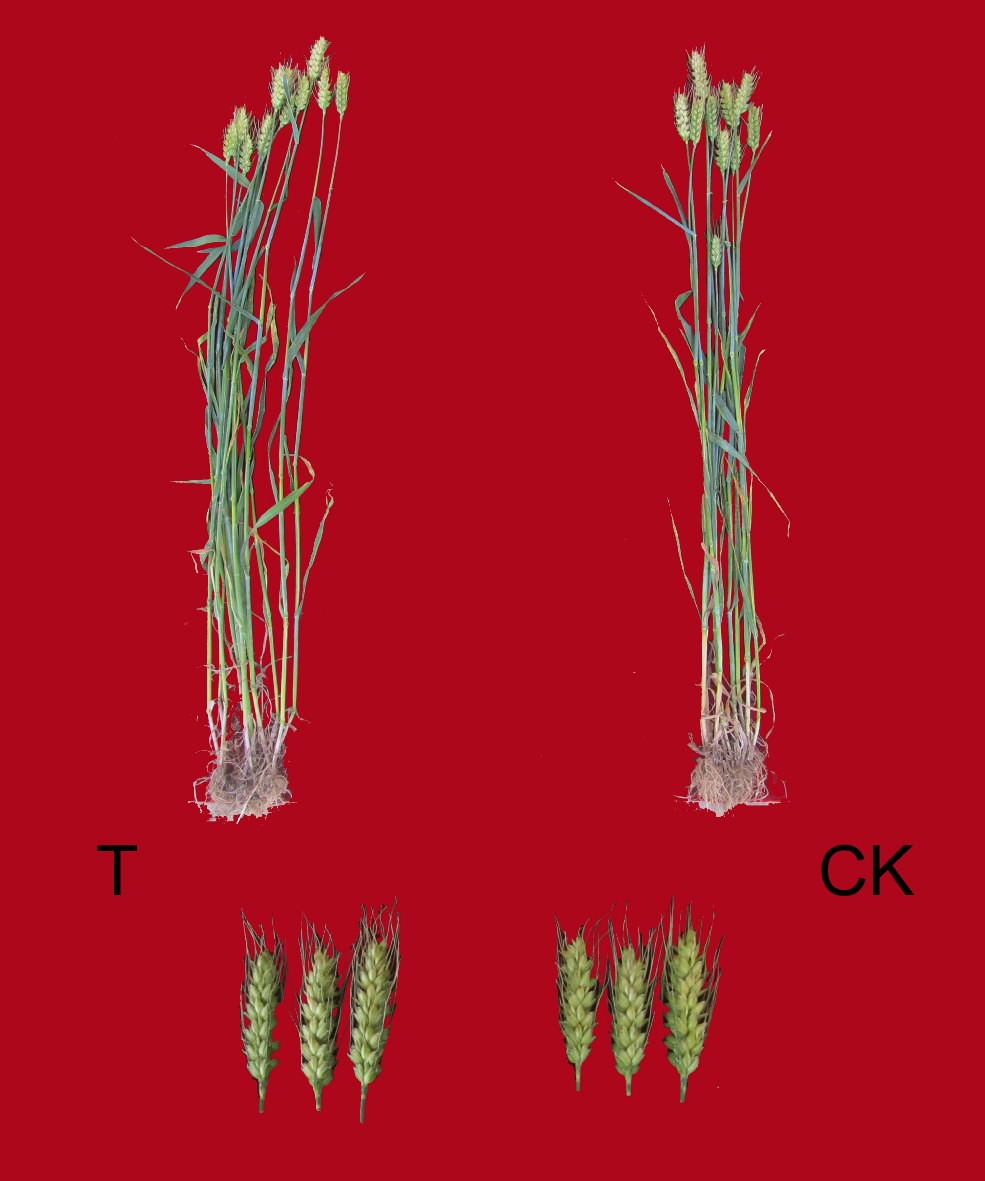

Supplement: Figure S2 — Phenotype characteristics of wheat plant under high N fertilizer(T) and normal N fertilizer (CK) conditions. [file Image2.JPEG]

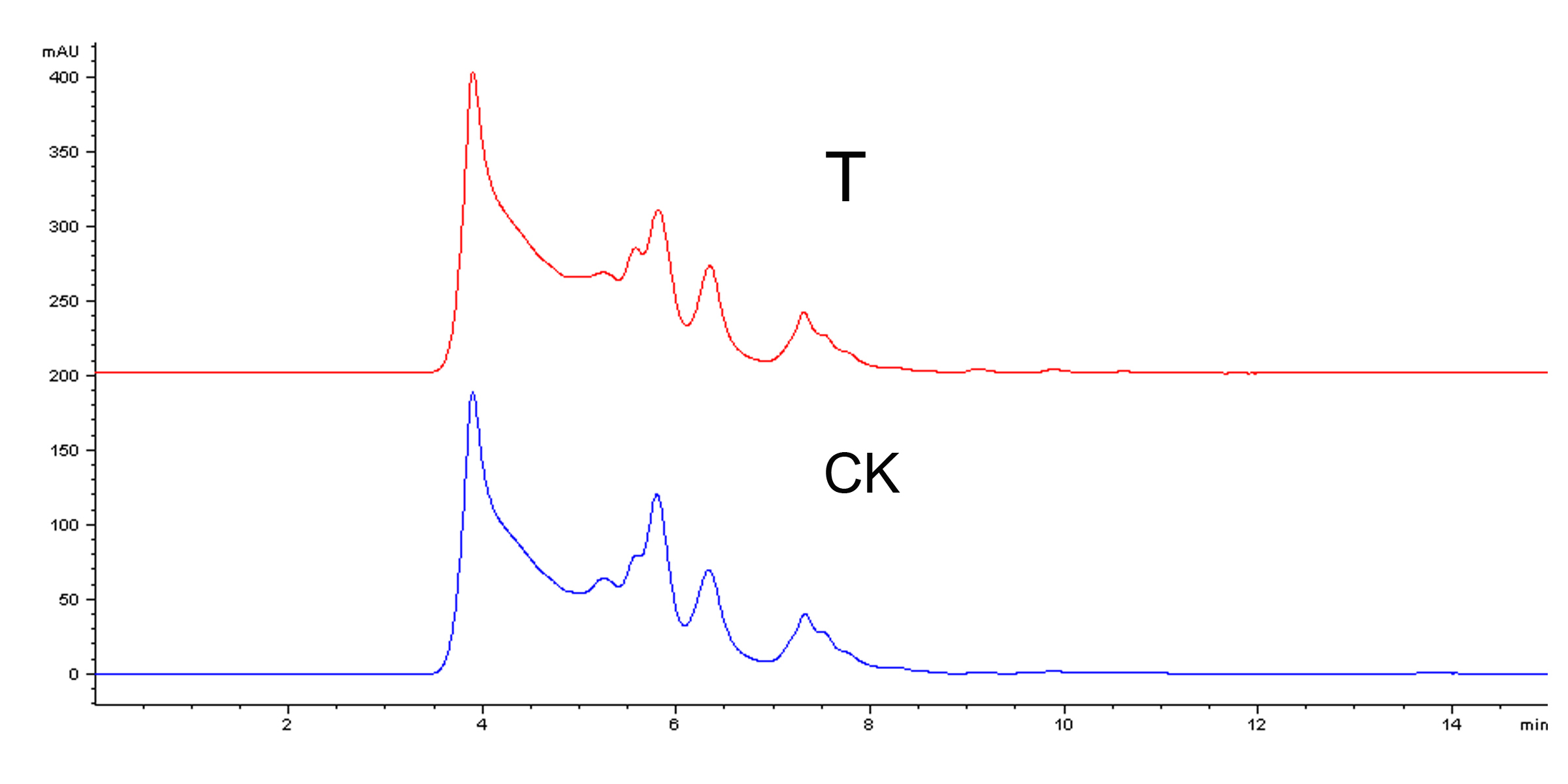

Supplement: Figure S3 — Image of SE-HPLC of wheat grains under high N fertilizer condition. [file Image3.JPEG]

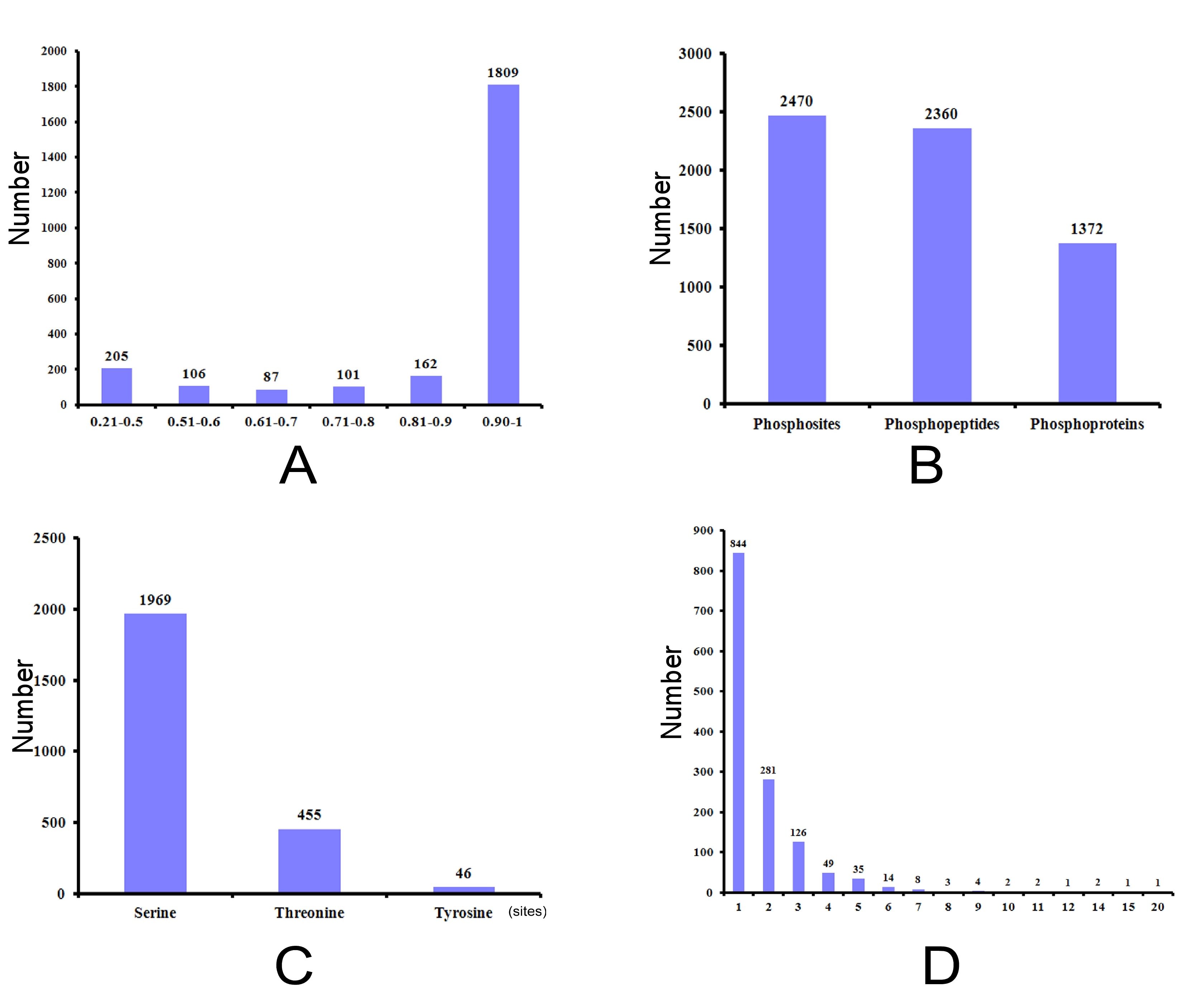

Supplement: Figure S4 — Phosphorylation status in the developing grains of Zhongmai 175 under high N fertilizer condition. (A) Number of phosphosites, unique phosphopeptides, and phosphoproteins identified in CK and treated groups. (B) Distribution of phosphosites on Ser, Thr and Tyr. (C) Distribution of phosphosites in each phosphoprotein. (D) Classification of phosphosites of all the identified sites in two groups. [file Image4.JPEG]

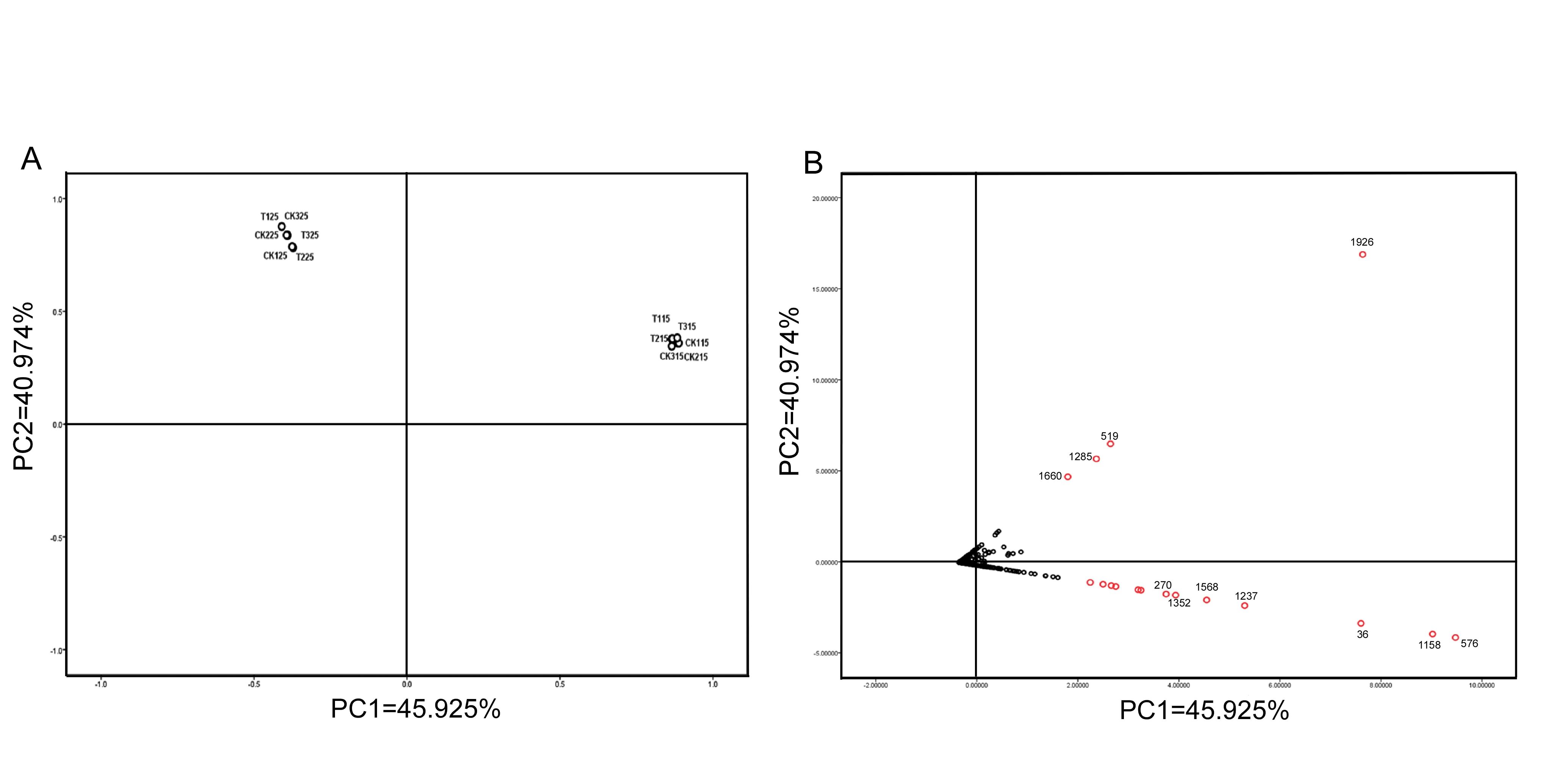

Supplement: Figure S5 — PCA analysis of the protein samples and phosphosites in CK and high N fertilizer groups. (A) PCA of individual protein samples in wheat grains under different N fertilizer conditions. (B) PCA of SCPL phosphosites spots in wheat grains under high N fertilizer. [file Image5.JPEG]

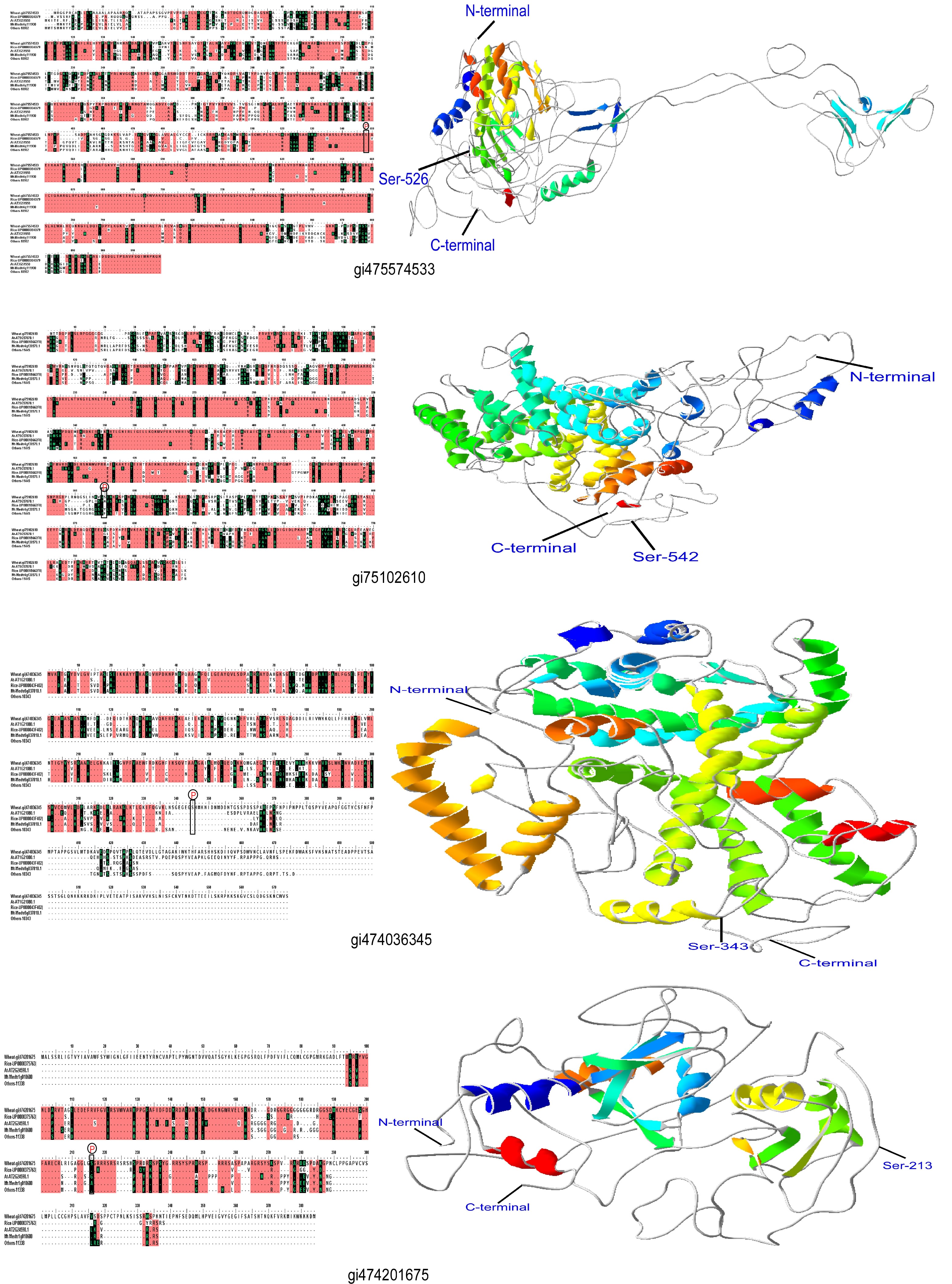

Supplement: Figure S6 — Sequence alignment and three-dimensional structure of four key phosphorylated proteins (gi475574533, gi75102610, gi474036345 and gi474201675). The phosphosites are marked with black box and highlighted in the 3D structure. [file Image6.JPEG]

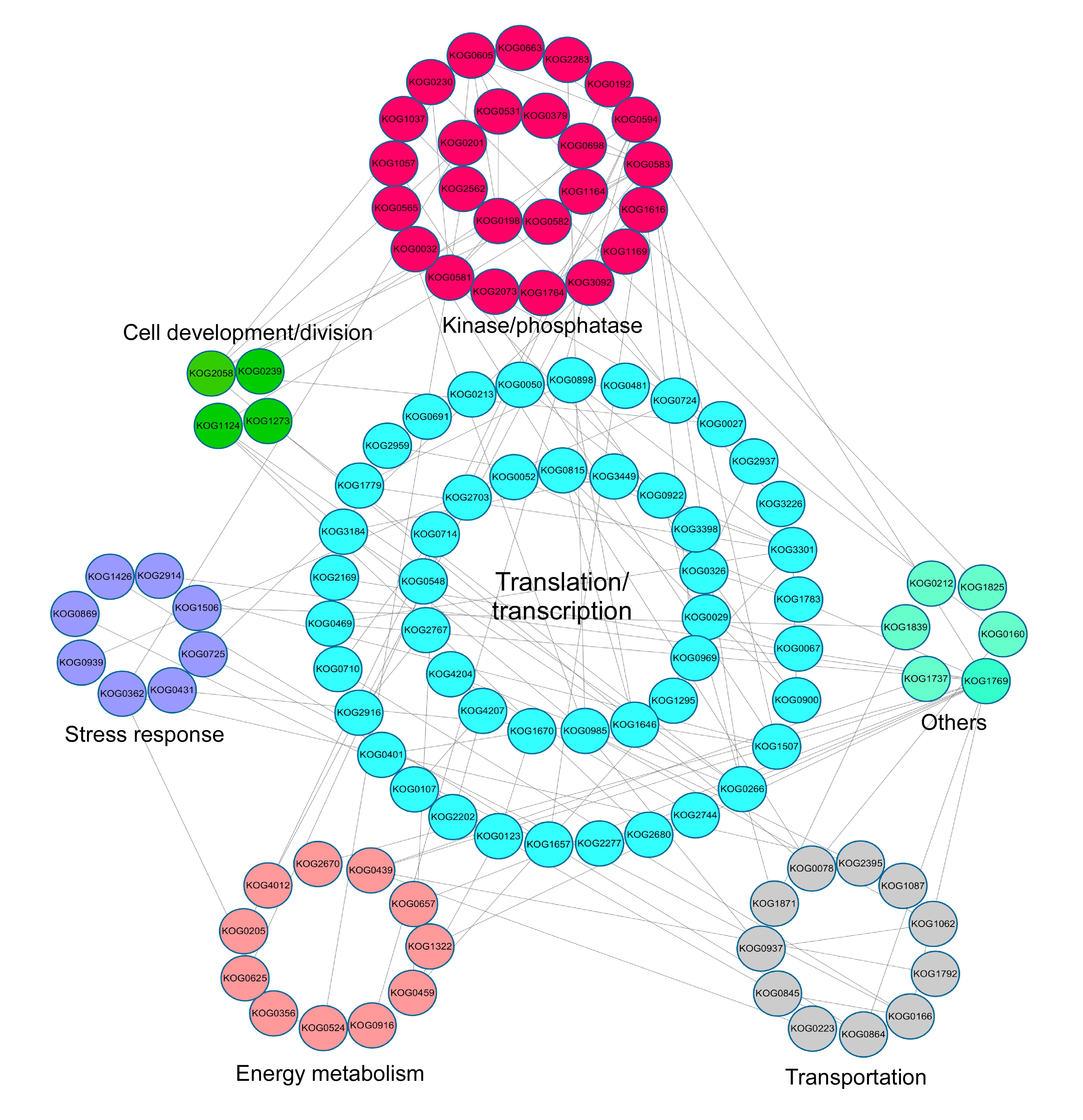

Supplement: Figure S7 — PPI analysis of all the identified phosphoproteins with SCPL under high N fertilizer condition. [file Image7.JPEG]
